# Accelerate Your Science: Direct-to-Biology Strategies in Medicinal Chemistry

**DOI:** 10.1021/acs.jmedchem.6c00996

**Published:** 2026-08-06

**Authors:** Kerstin Hiesinger, Stefan Knapp

**Affiliations:** † Institute of Pharmaceutical Chemistry, 9173 Goethe University Frankfurt, Max-von-Laue-Str. 9, Frankfurt am Main 60438, Germany; ‡ Structural Genomics Consortium (SGC), Buchmann Institute for Molecular Life Sciences (BMLS), Max-von-Laue-Str. 15, Frankfurt am Main 60438, Germany; § German Cancer Research Center (DKFZ), Im Neuenheimer Feld 280, Heidelberg 69120, Germany

## Abstract

Direct-to-biology (D2B) is a powerful strategy that accelerates early drug discovery. It enables compounds to be synthesized in miniaturized formats and evaluated directly as crude reaction mixtures. This bypasses the need for purification during the initial design-make-test cycle. Advances in robust synthetic methodologies, automation, reaction miniaturization, and biological screening have transformed D2B from a proof-of-concept approach into a versatile medicinal chemistry platform. This platform is applicable to fragment optimization, covalent ligands, macrocycles, proteolysis-targeting chimeras (PROTACs), molecular glues, and cellular phenotypic screening. This perspective focuses on the synthetic transformations, assay technologies, and platform implementations that drive modern D2B workflows. It emphasizes reaction robustness, assay compatibility, and practical implementation. Analysis of the current literature revealed that D2B is more governed by reaction reliability than synthetic diversity. Amide coupling and click chemistry dominate reported workflows, while more complex transformations remain underexplored. We discuss the complementary strengths and limitations of biochemical, biophysical, and cellular readouts, identify current bottlenecks in reaction scope and data management, and highlight emerging opportunities arising from reaction miniaturization, machine learning, automated experimentation, and advanced synthetic methodologies. Rather than replacing conventional medicinal chemistry, D2B fundamentally shifts experimental effort from purification toward early biological validation and is poised to become an integral component of future medicinal chemistry workflows.

## Graphical abstract

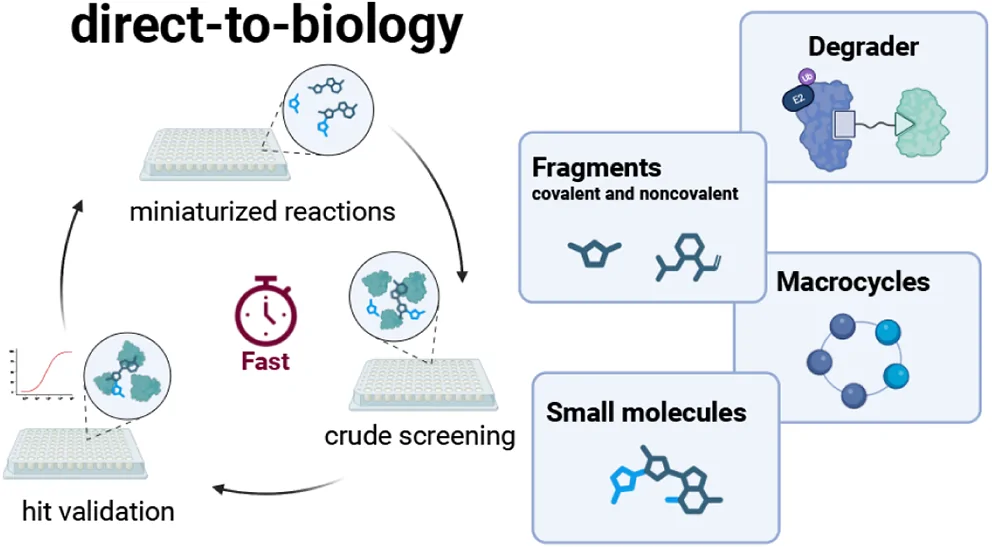

## Introduction

Early-stage medicinal chemistry is increasingly challenged by targets that demand rapid exploration of large, multidimensional chemical design spaces. This is particularly evident for emerging modalities such as proteolysis-targeting chimeras (PROTACs), molecular glues, covalent ligands, and fragment-based discovery, where hundreds to thousands of analogues may be required to identify productive combinations of warheads, linkers, exit vectors, and binding motifs. In these settings, the conventional design–make–purify–test paradigm frequently becomes rate-limiting, as purification and characterization of large numbers of speculative compounds consume substantial time and resources before their biological relevance has been established.

Direct-to-biology (D2B) approaches are changing this paradigm by integrating chemical synthesis and biological evaluation into a streamlined workflow. In a typical D2B campaign, compounds are synthesized in microscale or nanoscale plate-based formats, and crude reaction mixtures are screened directly in biochemical, biophysical, or cellular assays without prior purification. Active hits are subsequently resynthesized, purified, and validated, allowing rapid prioritization of relevant chemotypes while substantially reducing the number of compounds requiring conventional medicinal chemistry optimization (Figure 1C). D2B is particularly powerful for early hit identification and rapid structure–activity relationship (SAR) exploration, where the objective is to efficiently survey broad chemical space rather than immediately optimize lead-like properties. For example, D2B enables rapid assessment of linker, warhead, and exit-vector SAR in PROTACs, facilitates identification of productive electrophiles in covalent ligand discovery, and allows efficient prioritization of fragment elaboration strategies before committing to larger-scale synthesis.

**Figure 1:**
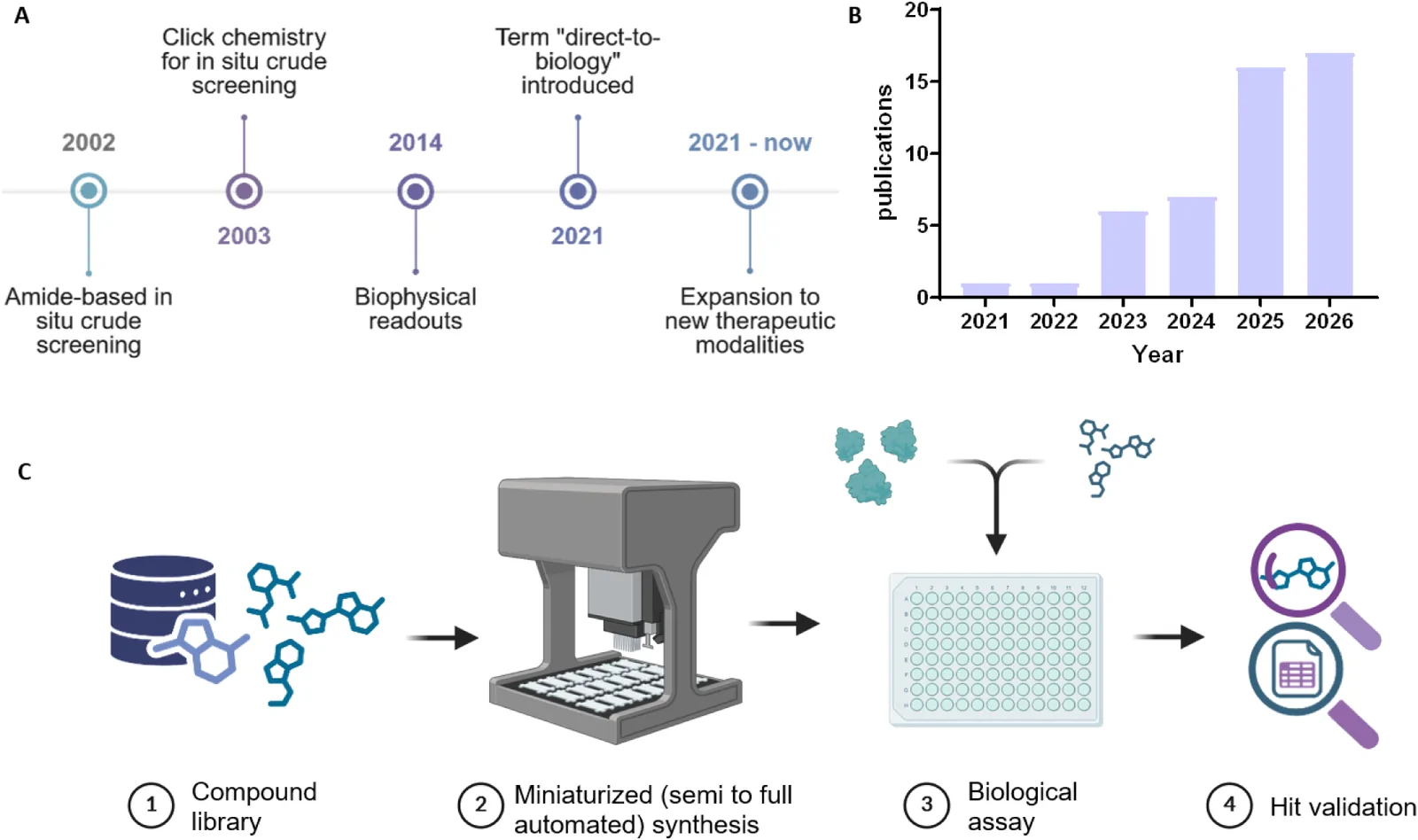
Historical development and general workflow of direct-to-biology (D2B). (A) Major milestones marking the transition from early in situ crude screening to modern D2B platforms. (B) Increasing numbers of D2B-related publications searching for the term “direct-to-biology”, illustrating the expansion of the field. (C) Schematic representation of a typical D2B campaign, in which compounds are synthesized in miniaturized plate formats, screened directly as crude reaction mixtures, and only validated hits are subsequently resynthesized and purified.

Although the term “direct-to-biology” has only recently gained widespread recognition,^1^ the underlying concept of screening crude reaction mixtures dates back more than two decades.^2–7^ As summarized in Figure 1A, the field has evolved from early amide-based in situ crude screening and the introduction of click chemistry toward biophysical screening platforms and, more recently, diverse therapeutic applications. This evolution has been enabled by the convergence of robust, modular, biology-compatible synthetic methodologies,^8^ reaction miniaturization, automated liquid handling, high-throughput analytical methods, and increasingly sophisticated biological assays, transforming D2B from isolated proof-of-concept studies into an increasingly adopted discovery platform in both academia and industry.

The rapid maturation of D2B is reflected by the steadily increasing number of publications in the field (Figure 1B). While recent reviews have summarized the emerging applications of D2B and the available chemical methodologies,^9,10^ the present review adopts a complementary perspective by focusing on the practical implementation of D2B platforms. Emphasis is placed on chemistry enabling D2B, the compatibility of synthetic transformations with direct biological evaluation, and the practical considerations that determine whether a reaction can be successfully translated into a robust D2B workflow.

Accordingly, this perspective focuses primarily on D2B methodologies reported since approximately 2014, marking the transition from early in situ screening studies to modern integrated D2B platforms driven by advances in automation, reaction miniaturization, and biophysical assay technologies. We discuss applications ranging from fragment-based discovery, covalent ligands, degraders, molecular glues, and macrocycles to biochemical, biophysical, and cellular screening platforms, while critically assessing current limitations and identifying opportunities for expanding the D2B reaction toolbox.

### D2B Using Biophysical Methods as Assay Readouts

Early D2B implementations demonstrated the value of biophysical measurements taken directly from crude reaction mixtures and proved that purification was not necessary to obtain reliable activity assessments. For instance, Murray and coworkers pioneered this approach by using surface plasmon resonance (SPR) to measure ligand dissociation rates directly from unpurified synthesis products.^11^ Because residence time is a key determinant of inhibitor efficacy, they first examined Heat Shock Protein 90 (HSP90) inhibitors derived from compound NVP-BEP800. However, library sizes were quite small in these initial studies. A small library of 13 analogues was generated via Suzuki coupling (PdCl_2_(PPh_3_)_2_, aq. NaHCO_3_, DMF, 3.5 mL) under microwave irradiation (10 min), followed by solvent removal and dissolution in DMSO (Scheme 1A). Crude samples **1** were diluted and subsequently analyzed by SPR using immobilized HSP90. Remarkably, dissociation constants differed by only ∼30% compared to purified compounds. The strategy was extended to a peptidylprolyl cis/trans isomerase, NIMA-interacting 1 (PIN1) inhibitor series **2** prepared by amide coupling followed by saponification (18.5 μL DMF matrix tubes; LiOH hydrolysis in MeOH/H_2_O for 4 h), after which crude products were analyzed by SPR. Off-rates from crude and purified samples agreed within ∼15%. Reagent carryover was evaluated by spiking purified compounds with typical reaction components, showing only minor effects on dissociation rates (5–18%), confirming assay robustness.

**Scheme 1:**
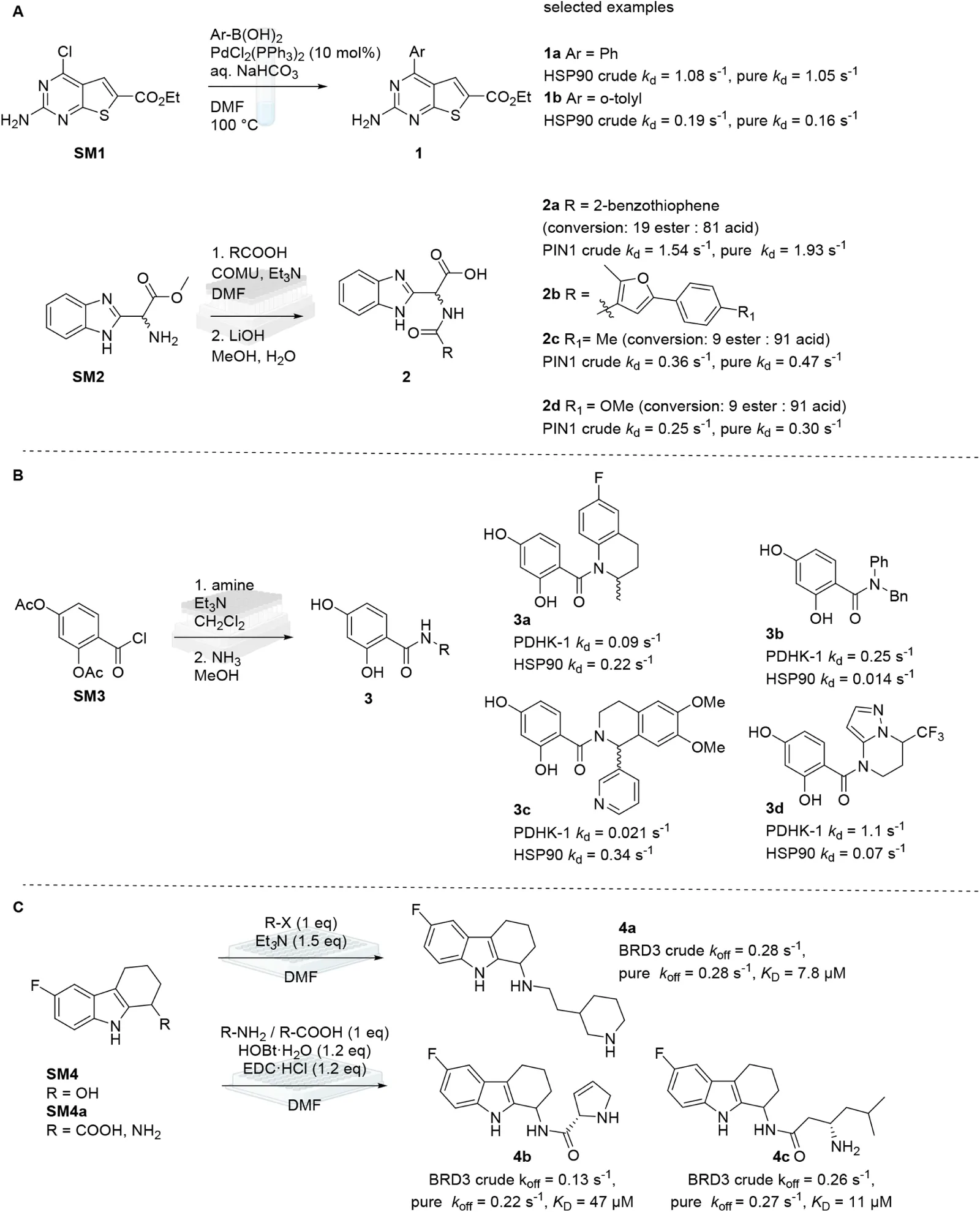
SPR-Based D2B Workflows^a^

a (A) Parallel synthesis of HSP90 inhibitors by Pd-catalyzed cross-coupling and amide coupling. (B) Off-rate screening of amide-based D2B libraries targeting PDHK1 and HSP90. (C) Fragment-to-lead optimization of BRD3 ligands using the REFiL platform and plate-based alkylation and amide coupling.

The same group later expanded this SPR-based off-rate screening (ORS) D2B workflow to resorcinol amide libraries **3** targeting the closely related GHKL ATPases pyruvate dehydrogenase kinase (PDHK) and HSP90 (Scheme 1B).^12^ Building on an initial fragment hit, secondary amines were coupled to a protected resorcinol acyl chloride **SM3** (10 μmol scale) in matrix tubes using triethylamine in DMF. After 26 h, 7 N ammonia was added to quench residual acyl chloride and regenerate the parent amide, followed by solvent removal (Genevac) and reconstitution in DMSO-*d*_6_. A total of 56 crude products were analyzed by LC–MS and screened directly by SPR against PDHK1 and HSP90. Three hits were resynthesized and purified, including chiral separation of compound **3a**, and a second D2B library delivered both PDHK-selective and HSP90-selective ligands (**3b**, **3c**, **3d**). In total, seven hits were identified, demonstrating that crude-product SPR can rapidly resolve selectivity and kinetic SAR at an early stage.

Adams and coworkers extended SPR-based D2B screening to fragment optimization through the development of Rapid Elaboration of Fragments into Leads (REFiL).^13^ Starting from a fragment **SM4** identified against the bromodomain-3 extra-terminal domain (BRD3-ET) (*K*_D_ = 380 μM), productive vectors for expansion were identified by evaluating commercial analogues before parallel alkylation and amidation libraries were prepared in 96-well plates (Scheme 1C). Reactions were conducted at 5 μmol scale (50 μL volume) and subjected to a standardized workflow involving solvent evaporation, optional LiOH hydrolysis or HCl/dioxane deprotection, filtration, and LC–MS analysis. Evaluation of the alkylation library showed that 50% of reactions produced the desired product at ≥10% purity, with a further 24% containing detectable product, whereas the amidation library proved more robust, with all 46 reactions yielding the desired amides and 96% achieving ≥10% purity. Crude reaction mixtures were screened directly by ORS,ORS), exploiting slower dissociation kinetics to identify higher-affinity products independent of exact conversion. A 92-member alkylation library and a 139-member amidation library yielded compounds with 8–17 μM affinity, representing up to a 33-fold improvement over the starting fragment. Resynthesized compounds (**4a**, **4b**, **4c**) showed good agreement between off-rates measured from crude and purified samples, validating the predictive value of ORS. While avoiding preparative purification, the workflow still relied on deprotection, filtration, and LC–MS quality control, placing REFiL at the interface between classical medicinal chemistry and modern D2B approaches.

In 2018, Gesmundo and coworkers introduced NanoSAR, integrating nanoscale synthesis with affinity-selection mass spectrometry (ASMS) for rapid SAR generation.^14^ Crude reaction mixtures were screened directly by ASMS, with stepwise dilution of target protein enabling ranking of ligands by competitive binding. Extracellular-signal-regulated kinase 2 (ERK2), MAPKAP-Kinase-2 (MK2), and Checkpoint-Kinase 1 (CHK1) served as targets. Reactions were performed at 1 μL scale in 1536-well plates using a Mosquito liquid handler and UPLC was used to assess conversion. Pooled crude products were incubated with protein and binders detected by HPLC–MS after size-exclusion separation and denaturation of protein–ligand complexes. An initial amide-coupled library (**5**) of 19 ERK2 inhibitors showed a strong correlation between NanoSAR rankings and functional bioassays (Scheme 2A). The platform was extended to nanoscale Suzuki and C–N couplings, screened under multiple conditions to ensure product **6** formation. Compounds synthesized under optimal conditions were resynthesized at larger scale and validated against MK2, confirming NanoSAR-predicted affinity trends. Finally, 3,114 reactions were performed using scaffold **SM7**, identifying productive conditions for 345 of 384 nucleophiles, with pooled crudes (**7**) screened against CHK1 across protein concentrations from 10 μM to 0.2 μM. Affinity rankings again matched purified IC_50_ values. Beyond ligand discovery, NanoSAR enabled rapid comparison of reaction classes and functional groups, revealing greater robustness for Suzuki couplings and deprioritizing motifs associated with consistently weak binding.

**Scheme 2:**
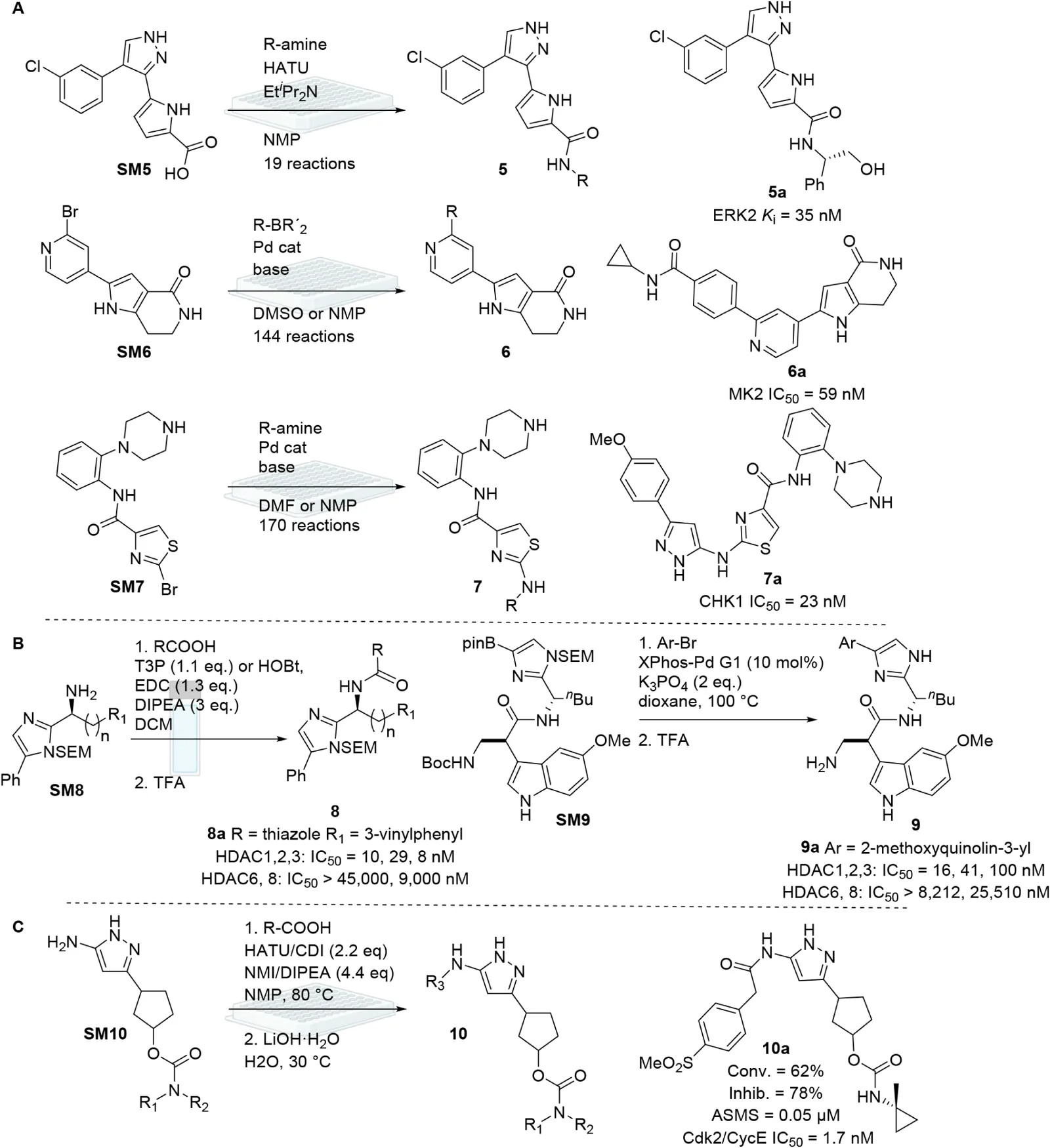
ASMS-Based D2B Workflows[Fn sch2-fn2]

a (A) NanoSAR workflow combining amide coupling and Pd-catalyzed transformations for ligand discovery against ERK2, MK2, and CHK1. (B) Affinity-selection mass spectrometry (ALIS) platform for HDAC ligand discovery. (C) UltraHTE amide coupling followed by bioluminescence-based screening and ASMS.

In 2024, Tyagarajan et al. reported a D2B workflow integrating affinity-selection mass spectrometry (ALIS) with early pharmacokinetic profiling to identify histone deacetylase (HDAC) inhibitors (Scheme 2B).^15^ ALIS combines 2D chromatography with high-resolution MS to rank binding directly from crude mixtures, while microsomal intrinsic clearance (Cl_int_) was assessed in parallel by MS. Starting from a pan-HDAC inhibitor, 45 analogues incorporating zinc-binding and nonzinc-binding motifs were synthesized via amide coupling (from **SM8** to **8**) and Suzuki–Miyaura cross-coupling (from **SM9** to **9**), followed by TFA deprotection (1-dram vials, 0.5 mL solvent). After Genevac evaporation, residues were dissolved in DMSO and were screened directly by ALIS for HDAC2 binding and microsomal stability; remaining material was purified for confirmation. Relative ALIS affinities correlated well with enzymatic IC_50_ values in the first series and reliably distinguished strong from weak binders in the second. Crude and purified Cl_int,u_ values were comparable, indicating tolerance to residual palladium. Compound prioritization using Lipophilic Ligand Efficiency (LLE) and Lipophilic Metabolism Efficiency identified multiple candidates (e.g., **8a**, **9a**) with LLE ≥ 4, illustrating how D2B platforms can simultaneously optimize potency and metabolic stability.

Bentley and coworkers reported a D2B crystallographic fragment-expansion campaign targeting *E. coli* Disulfide bond-formation protein (DsbA),^16^ building on an earlier fragment hit.^17^ Fragment optimization yielded a diaryl ether acid (**SM11**, *K*_D_ = 490 μM), which was diversified via plate-based amide coupling with 93 amines (Scheme 3A). After screening multiple conditions, HATU (1.5 equiv amine, MeCN, RT) was selected, giving 20–85% conversion depending on amine class. Compatibility with crystallographic soaking was evaluated, revealing that COMU reduced diffraction quality (2.4 Å) relative to HATU or EDC (1.9–1.8 Å). Reactions were performed at 2 μmol scale in 96-well plates; LC–MS confirmed product formation of **11** in 65/93 reactions, with partial conversion in 18 additional wells. Crude DMSO stocks were soaked directly into EcDsbA crystals, yielding 90 data sets (1.8–2.5 Å). Electron density for the parent fragment was observed in 82 structures, while four data sets revealed density consistent with amide-expanded analogues. Resynthesized hits were validated by ^1^H–^15^N HSQC NMR, with a pyrazole analogue **11b** reaching *K*_D_ = 62 μM. Even low-conversion reactions (∼6%) produced detectable density, although residual coupling reagents diminished map quality.

**Scheme 3:**
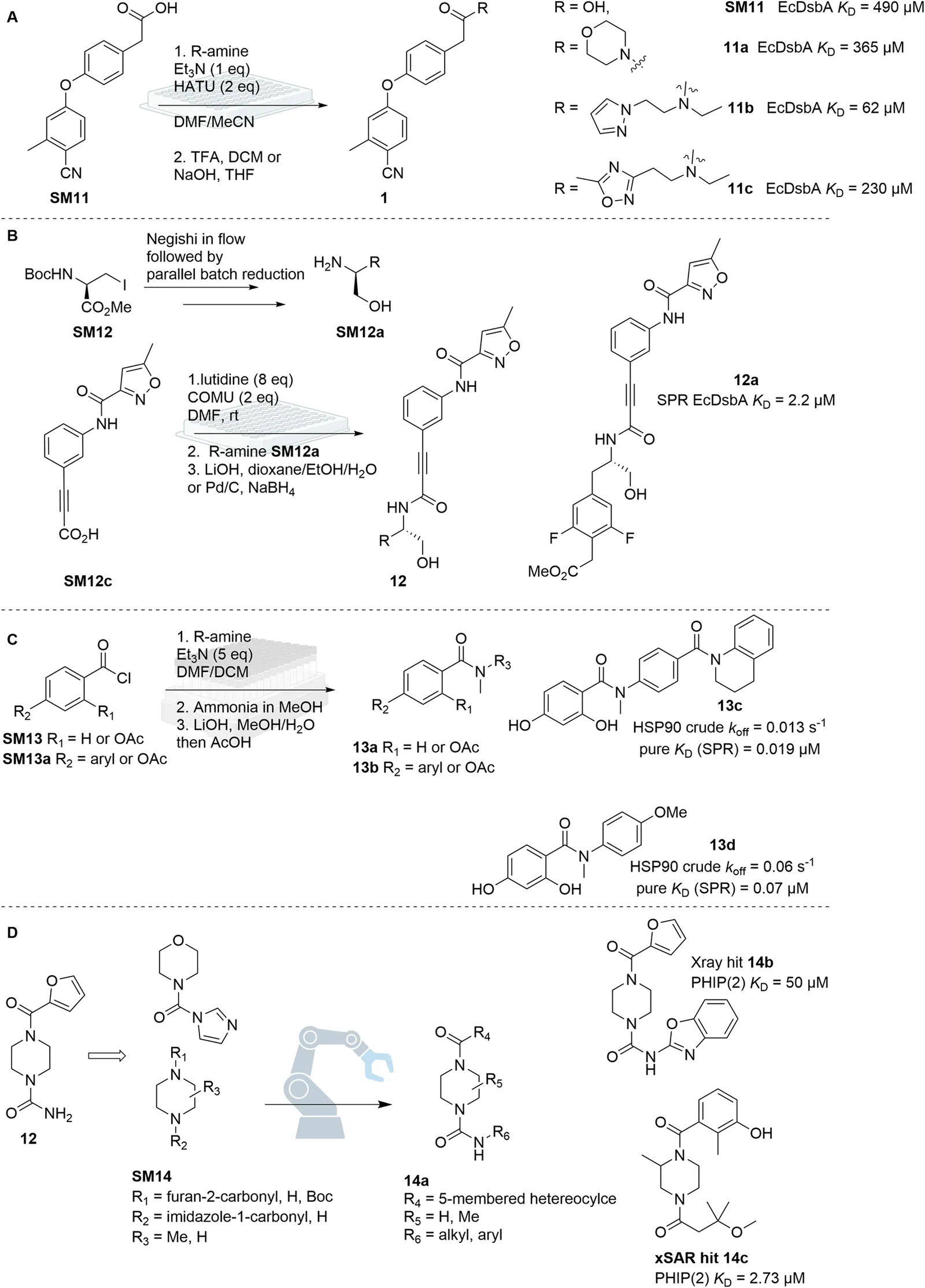
D2B Workflows Employing X-ray Crystallography[Fn sch3-fn3]

a (A,B) Plate-based amide coupling followed by direct X-ray screening for fragment optimization. (C) Amide coupling-based D2B workflow for HSP90 ligand discovery. (D) B-SPA workflow combining automated multistep synthesis with crystallographic screening of PHIP(2) analogues.

In a second report, they report a structure-guided D2B strategy to target a cryptic pocket in *E. coli* DsbA and generate dual-site antivirulence inhibitors.^18^ Starting from a weak fragment (*K*_D_ = 1070 μM), linker growth into the adjacent hydrophobic groove was guided by X-ray crystallography, SPR, ITC, and ^15^N–^1^H HSQC, identifying an α-substituted hydroxyethylamide motif that improved binding (e.g., (*S*)-phenylalaninol, *K*_D_ = 44 μM). Further optimization employed a telescoped D2B workflow integrating automated flow chemistry, microscale plate synthesis, and crude screening (Scheme 3B). A library of aryl-substituted analogues was generated via flow Negishi coupling using an activated Zn column (22 g, 60 °C, 2 bar, DMF), with organozinc formation verified by iodine test and nine reactions run per cycle (∼6 h). Reaction mixtures were minimally worked up (metal scavenging, Celite filtration) and directly advanced through NaBH_4_ reduction (0 °C to RT), TFA deprotection, and strong cation exchange (SCX) purification to yield amino alcohol intermediates **SM12a** without chromatographic purification. Final analogues were assembled by microscale amide coupling (COMU, lutidine, DMF, 96-well format, overnight), followed by optional ester hydrolysis (LiOH) or Cbz deprotection (Pd/C, NaBH_4_), generating 92 crude products **12**. Crude libraries were screened directly using orthogonal methods: SPR off-rate screening and ASMS, enabling hit identification independent of exact concentration. Resynthesis validated hits, with analogue **12a** showing low micromolar affinity (SPR *K*_D_ = 2.2 μM; ITC *K*_D_ = 1.5 μM) and enzymatic inhibition (IC_50_ = 30 μM). Structural data revealed conserved cryptic-pocket binding with flexible adaptation in the hydrophobic groove. Compounds reduced bacterial motility without affecting growth, consistent with antivirulence DsbA inhibition, demonstrating efficient fragment-to-lead optimization via D2B.Baker

Baker and colleagues applied a D2B X-ray crystallography workflow to fragment expansion against HSP90 and PDHK2.^19^ Known ligands (**SM13**, **SM13a**) were diversified via amide coupling with diverse amines in matrix tubes, followed by optional deprotection, with conversion assessed by LC–MS (Scheme 3C). Crude (and matched purified) products **13a**, **13b** were soaked into protein crystals (2 mM, triplicate, at multiple time points). Because HSP90 crystals were incompatible with the XChem sitting-drop format, hanging-drop crystals were manually transferred for soaking. Across 234 data sets, optimal ligand density was obtained after 2 days of soaking. Electron density was observed for 11/83 crude amides with PDHK2 but only one with HSP90. Data were analyzed manually and using the software PanDDA,^20^ and SPR confirmed affinity gains for identified hits. The authors also provided a detailed analysis of target-specific false-negative rates and noted frequent binding of starting materials as well as empty binding sites. Both reports highlight that X-ray-based D2B approaches can provide valuable structural insights but should be complemented by biophysical assays to guide hit prioritization and optimization.

The limitations observed in the HSP90 and PDHK2 studies, particularly false negatives and target-dependent hit rates, became a central focus of later crystallography-driven D2B workflows. Building on these concepts, von Grosjean and coworkers developed Binding-Site Purification of Actives (B-SPA), a platform combining automated multistep synthesis with direct screening of crude reaction mixtures by high-throughput X-ray crystallography against the bromodomain of Pleckstrin Homology Domain-Interacting Protein 2 (PHIP(2)).^21^ Using an OpenTrons liquid-handling robot, six telescoped synthetic routes comprising one- to five-step sequences were implemented in 2 mL 96-well plates at scales of approximately 20–400 μmol. Across 1876 fragment elaboration attempts involving 6592 reaction steps, LC–MS confirmed product formation in 1108 reactions, generating 969 crystallographic data sets and yielding 22 ligand-bound crystal structures (Scheme 3D). Structural analysis established SAR around the fragment **14** scaffold and identified both conserved and alternative binding modes, while orthogonal biophysical assays confirmed progression to low-micromolar affinity (**14b**, IC_50_ = 31.15 μM, *K*_D_ = 50.03 μM). Although crude reaction mixtures **14a** were screened directly, the workflow incorporated extensive automated solvent exchanges, extractions, deprotections, and workup operations, placing it at the boundary between classical D2B and highly automated parallel medicinal chemistry. Importantly, several analytically confirmed products failed to generate crystallographic binding events, indicating that negative crystallographic outcomes did not necessarily reflect a lack of target engagement. Motivated by these observations, the authors subsequently reanalyzed the PHIP(2) data set using ligand-based crystallographic SAR (xSAR).^22^ The model recovered previously missed binders and was prospectively applied to prioritize compounds from the Enamine REAL database.^23^ Crystallographic evaluation of purchased compounds identified nine confirmed binders (9.68% hit rate, e.g., **14c**), demonstrating that large D2B crystallographic data sets can be transformed into predictive design tools while highlighting the challenge of readout-dependent false negatives.

Douthwaite and colleagues developed a nanoscale D2B platform for discovering cyclin-dependent kinase 2/cyclin E (Cdk2/CycE) inhibitors, integrating ultrahigh-throughput experimentation (ultraHTE), biochemical screening, ASMS, X-ray crystallography, and cellular profiling (Scheme 2C).^24^ A library of 768 compounds was generated from six amino-pyrazole cores (**SM10**) and 128 carboxylic acids via a two-step amide coupling/deprotection sequence optimized for D2B compatibility using NMP and aqueous LiOH. Reactions were conducted in 1536-well plates at a 100 nmol scale (1 μL), with four conditions per target (HATU or CDI and DIPEA or NMI). UPLC–MS confirmed the successful synthesis of 691 compounds **10** (90%). ADP-Glo assay compatibility was validated, with minor background inhibition from starting materials (<15%), while a Caliper assay was excluded due to poor correlation. ADP-Glo screening showed ∼450 compounds with ≥80% inhibition, necessitating ASMS-based ranking. Optimized pooling and detection enabled efficient deconvolution, with 103 compounds tested and 12 yielding quantitative affinity data. Selected compounds were resynthesized (50 μmol), confirming agreement with IC_50_ values. Five crude representatives (e.g., **10a**) from different acid families were advanced to crystallography, all yielding ligand-bound structures and revealing diverse binding modes. Cellular activity was assessed via 5-ethynyl-2′-deoxyuridine (EdU) incorporation in HCT-116 cells, showing dose-dependent inhibition consistent with G0/G1 arrest. By integrating synthesis, biochemical screening, affinity ranking, crystallography, and cellular phenotyping within a single miniaturized workflow, this study demonstrates how D2B can compress large portions of the hit-to-lead optimization process into a single experiment and represents one of the most comprehensive examples of an ultraHTE-enabled D2B platform reported to date.

Grant and coworkers developed a photoreactive fragment discovery platform based on diazirine- and tetrazole-derived photoactivatable handles that enables direct screening of unpurified compounds by intact-protein mass spectrometry. In their initial work, libraries of diazirine-containing fragments (PhABits **15**) were incubated with purified proteins to allow reversible binding, followed by UV irradiation (302 nm) to generate short-lived carbene intermediates that covalently trap proximal residues, enabling detection of transient interactions by intact-protein LC–MS.^25^ This established photo-cross-linking MS as a tag-free fragment screening strategy compatible with native protein conformations. The platform was subsequently transformed into a D2B workflow by generating photoreactive probes in plate and screening crude reaction mixtures directly (Scheme 4A).^1^ A diazirine *N*-hydroxysuccinimide (NHS) ester was coupled to a preselected alkylamine library via succinimide-activated amide formation in 384-well plates (100 mM, 80 μL DMSO, *N*-ethylmorpholine, RT). Poor conversion with anilines led to their exclusion, while LC–MS analysis showed successful labeling for 980 of 1073 amines, with primary and secondary amines performing best. Residual NHS ester caused nonspecific protein labeling, prompting the introduction of a hydroxylamine-based quencher (2.2 equiv, 1 h) to ensure assay compatibility. Crude products were screened directly against human carbonic anhydrase I (CAI) at a concentration of 100 μM, followed by UV irradiation and intact-protein LC–MS analysis. Seven hits (>1.5% cross-linking) were validated by competition with a zinc-binding ligand, and purified compounds confirmed crude screening trends, revealing sulfonamides as a privileged motif (**15a**). Iterative D2B cycling guided by similarity analysis substantially increased hit rates.

**Scheme 4:**
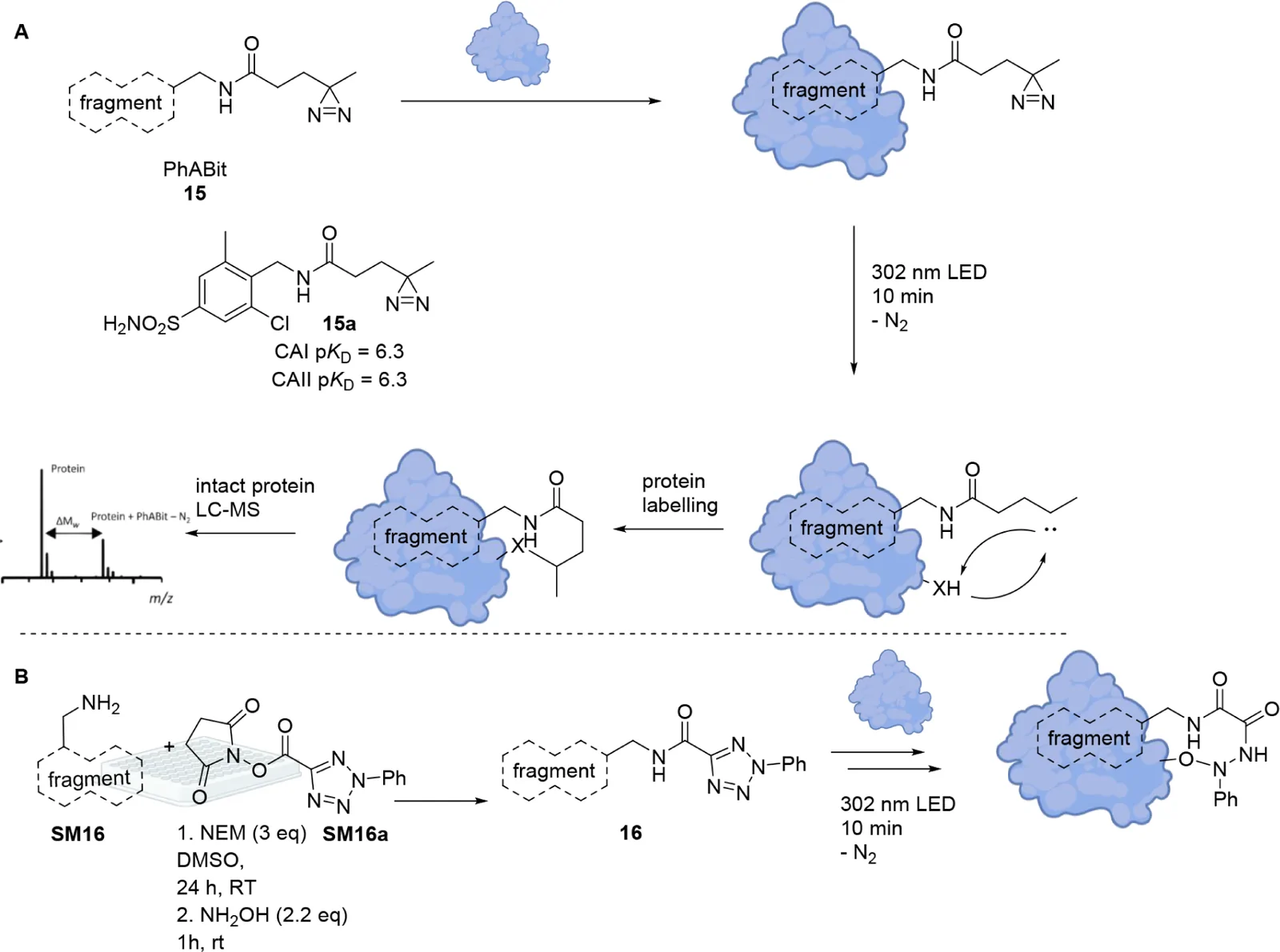
D2B Workflows Employing Intact-Protein LC–MS[Fn sch4-fn4]

a (A) PhABit platform for photoreactive fragment screening and hit identification by intact-protein LC–MS. (B) Evolution of the PhABit platform through the incorporation of ACTs as photoactivatable handles to improve crosslinking efficiency.

Developing the method further, the authors replaced diazirines with 2-aryl-5-carboxytetrazoles (ACTs) as photoactivatable handles (Scheme 4B).^26^ Upon UV irradiation, ACTs generate longer-lived nitrilimine intermediates that couple with carboxylic acids to form 1,2-diacylhydrazines, improving cross-linking efficiency. Using succinimide-ester-mediated amide coupling in 384-well plates, 546 reactions were assembled, with 496 confirmed by LC–MS after hydroxylamine quenching. The crude library **16** was screened by intact-protein MS against bromodomain-containing protein 4 (BRD4)-BD1, B-cell lymphoma 6 (BCL6), CAI/CAII, and KRAS^G12D^, yielding target-specific hits across all targets. Buffer optimization identified HEPES as optimal to reduce nonspecific labeling. To further improve protein integrity and signal quality, irradiation conditions were refined using photosensitizers, with benzophenone at 365 nm providing improved cross-linking (18%) under milder conditions.

Collectively, these studies highlight that the major biophysical D2B readouts fulfill distinct rather than interchangeable roles. SPR-based off-rate screening provides robust, concentration-independent kinetic ranking and is particularly well suited for rapid SAR generation from crude reaction mixtures. ASMS efficiently enriches the highest-affinity ligands from very large libraries despite variations in reaction conversion, making it an attractive primary screening method for ultrahigh-throughput D2B campaigns. In contrast, X-ray crystallography offers unrivaled structural insight by directly revealing binding modes and enabling structure-guided design. However, this technology does not provide information on binding affinity and it requires a substantial experimental effort. Moreover, target-dependent hit rates and readout-dependent false negatives limit its effectiveness as a primary screening method. Consequently, crystallographic D2B is most powerful when integrated with orthogonal biophysical assays, using SPR or ASMS for hit prioritization and affinity assessment, while exploiting X-ray crystallography to guide subsequent medicinal chemistry optimization.

### D2B Using Enzyme Kinetic Readouts

Jing and coworkers developed a series of plate-based Cu-catalyzed azide–alkyne cycloaddition (CuAAC) workflows enabling rapid diversification of alkyne-functionalized scaffolds with azide libraries and direct screening of crude reaction mixtures across multiple targets. Their initial implementation targeted cell division cycle 25 phosphatase (Cdc25),^27^ where quinone-based inhibitors (derived from NSC663284 **17a**, NSC668394 **17b**, and **17c**) were functionalized with alkynes and reacted with azides in 96-well plates (0,5 μmol, 100 μL raction volume) (Scheme 5A). Reaction progress was monitored by TLC, and crude mixtures were diluted and screened at 10 μM against all Cdc25 isoforms using a fluorometric phosphatase assay based on cleavage of 3-*O*-methylfluorescein phosphate. This workflow delivered compound **17d** with up to 9-fold improved potency compared with the starting point **17a**, and the synthesized compounds retained antiproliferative activity in cells. The platform was expanded in 2021 by introducing parallel amide coupling (HBTU/HOBt/DIEA, DMF, 96-well format) to complement CuAAC diversification.^28^ Reactions (4 mM acid, 4.4 mM amine, 0.1 equiv HBTU/HOBt, 3 equiv DIEA, 100 μL, 24 h or previously established CuAAC reaction) were monitored by mass analysis and TLC, and crude products were evaluated in Cdc25 inhibition and cell viability assays, identifying compound **17e** as the most active compound.

**Scheme 5:**
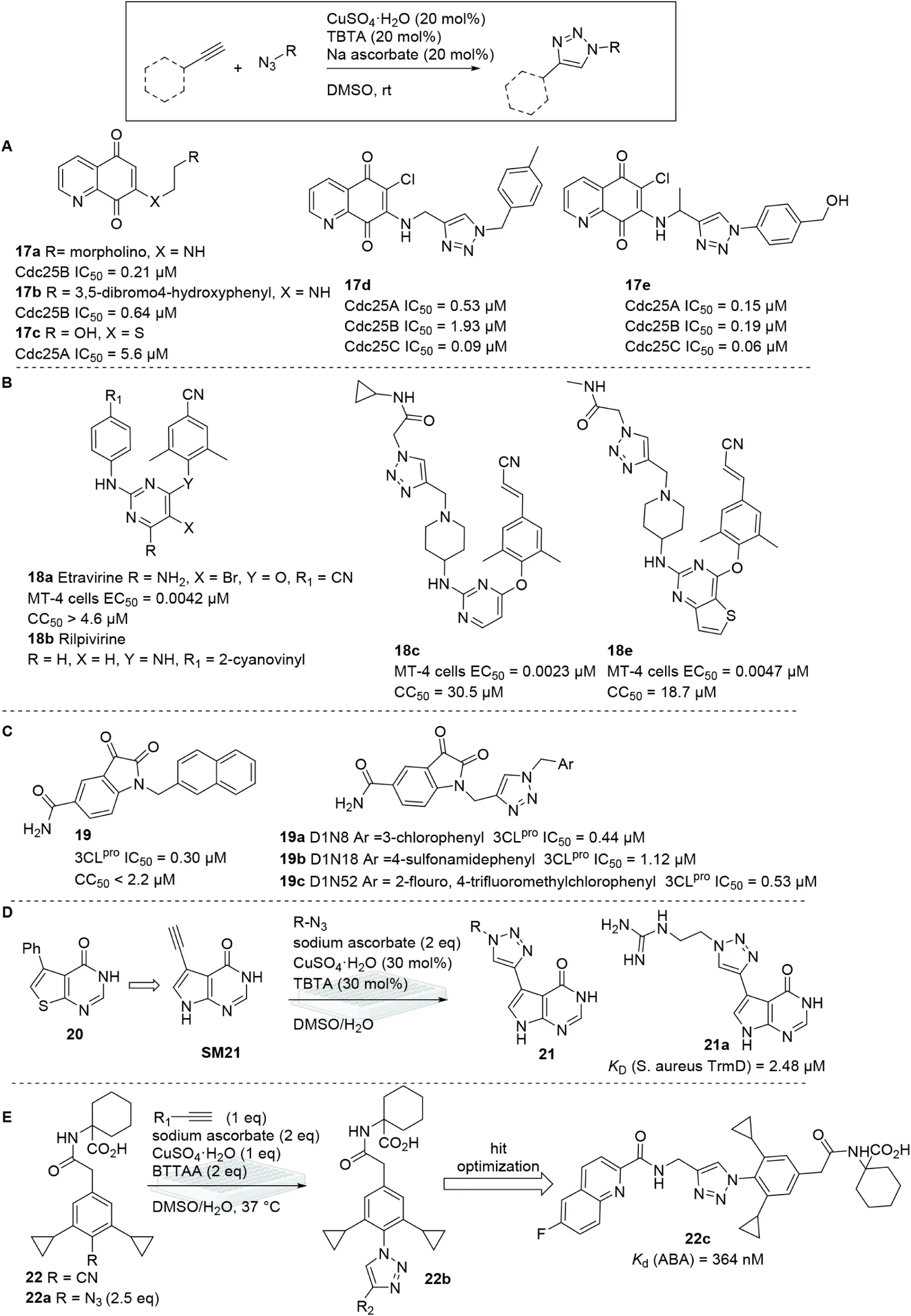
D2B Workflows Using CuAAC Chemistry[Fn sch5-fn5]

a (A) Parallel synthesis of Cdc25 inhibitors followed by fluorometric phosphatase screening. (B) Discovery of non-nucleoside reverse transcriptase inhibitors. (C) Identification of SARS-CoV-2 3CL^pro^ inhibitors. (D) Discovery of TrmD inhibitors using a fluorescence polarization assay established in the same study. (E) Extension of D2B beyond classical medicinal chemistry through the discovery of antagonists of the abscisic acid receptor.

Building on this chemistry–biology workflow, Kang and coworkers applied CuAAC-based screening to HIV-1 non-nucleoside reverse transcriptase inhibitors (NNRTIs).^29^ Six alkyne-functionalized cores were combined with 39 azides to generate 156 crude triazoles, which were screened at 267 nM in an ELISA-based reverse transcriptase inhibition assay, with CuSO_4_/TBTA/sodium ascorbate controls. Twenty-two hits exceeded reference inhibitors and were resynthesized and purified, followed by antiviral evaluation in MT-4 cells infected with wild-type and resistant HIV-1 strains, with cytotoxicity assessed by an MTT assay. Lead compounds reached EC_50_ values of 3 nM against the wild-type virus. In a subsequent iteration,^30^ alkyne-modified etravirine/rilpivirine scaffolds were diversified with 49 azides, yielding **18c** (EC_50_ of 2–9 nM against mutants), which was further optimized by classical SAR to compound **18e**, achieving potency comparable to etravirine (**18a**) against resistant HIV-1 strains, with cytotoxicity assessed by an MTT assay (Scheme 5B). Most recently, the platform was extended to SARS-CoV-2 3CL protease (3CL^pro^).^31^ An alkyne-modified **19** scaffold was coupled with 58 azides, with representative crude mixtures characterized by LC–MS (Scheme 5C). Crudes were screened directly in a FRET-based 3CL^pro^ enzymatic assay, identifying three hits with inhibition comparable to **19**. Purified compounds were evaluated in dose–response and cell-based antiviral assays, revealing reduced cytotoxicity (CC_50_ > 20 μM) relative to **17**, albeit with limited antiviral efficacy (compounds **19a**-**19c**).

Hübner and coworkers applied a D2B workflow to discover inhibitors of *Staphylococcus aureus* tRNA m^1^G37 methyltransferase (TrmD) (Scheme 5D).^32^ Building on thienopyrimidinone ligands (**20**) targeting the *S*-adenosyl-methionine (SAM) binding pocket, representative compounds showed micromolar binding by ITC (*K*_D_ = 12.9–19.8 μM), motivating the development of a fluorescence polarization (FP) displacement assay using a SAM-pocket tracer. CuAAC was selected for nanoscale synthesis after pilot reactions showed >90% conversion, and crude products displayed comparable affinities to purified material. Alkyne-functionalized pyrrolo­[2,3-*d*]­pyrimidinones **SM21** were coupled with a 320-member azide library in 96-well plates (96 nmol scale, CuSO_4_·5H_2_O/TBTA/sodium ascorbate, DMSO/H_2_O, 16 h), with LC–MS confirming high conversion. Crudes **21** were screened directly at 1 μM in the FP assay, identifying five near-complete displacers. Resynthesized hits (e.g., **21a**) showed *K*_D_ values of 0.72–2.48 μM, with selectivity confirmed across TrmD isoforms and subsequent optimization yielding a selective prodrug.

Illustrating that D2B concepts can be applied beyond classical medicinal chemistry and therapeutic drug-discovery targets, Vaidya and coworkers reported a click chemistry-enabled D2B strategy for the discovery of antagonists of abscisic acid (ABA, plant hormone) receptors.^33^ Starting from the synthetic ABA agonist opabactin (**22**), the authors designed an azide-containing intermediate (**22a**) that enabled rapid diversification through CuAAC (Scheme 5E). A library of 4002 commercially available alkynes was reacted with **22a** in 96-well plates using standardized click conditions. Reactions were performed on a 100 nmol scale in a total volume of 20 μL per well and incubated at 37 °C for 48 h. Prior to screening, the authors demonstrated that residual copper, ligands, reductants, and other reaction components did not significantly interfere with the biological assay, enabling direct evaluation of unpurified reaction mixtures. The crude reactions **22b** were diluted 100-fold and screened in a receptor-mediated phosphatase recovery assay that monitored disruption of interactions between ABA receptors and Protein Phosphatase 2C (PP2C), the principal negative regulator of ABA signaling. Screening identified 204 active reactions (≈5% hit rate), with significant enrichment of peptidotriazole-containing products. Subsequent focused optimization yielded antabactin (**22c**), a potent pan-ABA receptor antagonist with picomolar-to-low nanomolar receptor affinity.

Chen and coworkers reported a microplate-based parallel synthesis and in situ screening workflow to rapidly optimize celastrol toward selective peroxiredoxin-1 (PRDX1) inhibitors.^34^ Two complementary libraries were constructed directly in 96-well plates by modifying the C-20 position of celastrol via EDCI/OxymaPure/NMM-mediated amide coupling (Lib-C1, 1360 members) and Curtius rearrangement–derived isocyanate followed by urea formation (Lib-C2, 1360 members; 50 μL per well), yielding a 2720-compound library **23** assembled at ∼10 mM theoretical concentration under semiautomated handling (Scheme 6A). The reactions were compatible with direct biological testing, with selected wells analyzed by UPLC–MS to confirm product formation. Crude mixtures were evaluated using an in situ PRDX1 peroxidase assay based on a Trx–TrxR–NADPH coupling system, in which oxidized peroxiredoxins are efficiently reduced and enzyme activity is monitored by measuring NADPH oxidation at 340 nm, identifying 126 primary hits and narrowing to 22 compounds after a second screening round. These were resynthesized and purified, with most showing submicromolar PRDX1 inhibition (IC_50_ = 0.041–0.179 μM). Lead compound **23a** displayed an IC_50_ of 0.042 μM, representing a 4.6-fold improvement over celastrol, and exhibited strong selectivity across the PRDX family (PRDX2/3/4/6 IC_50_ > 50 μM) along with good antiproliferative activity in colorectal cancer cells.

**Scheme 6:**
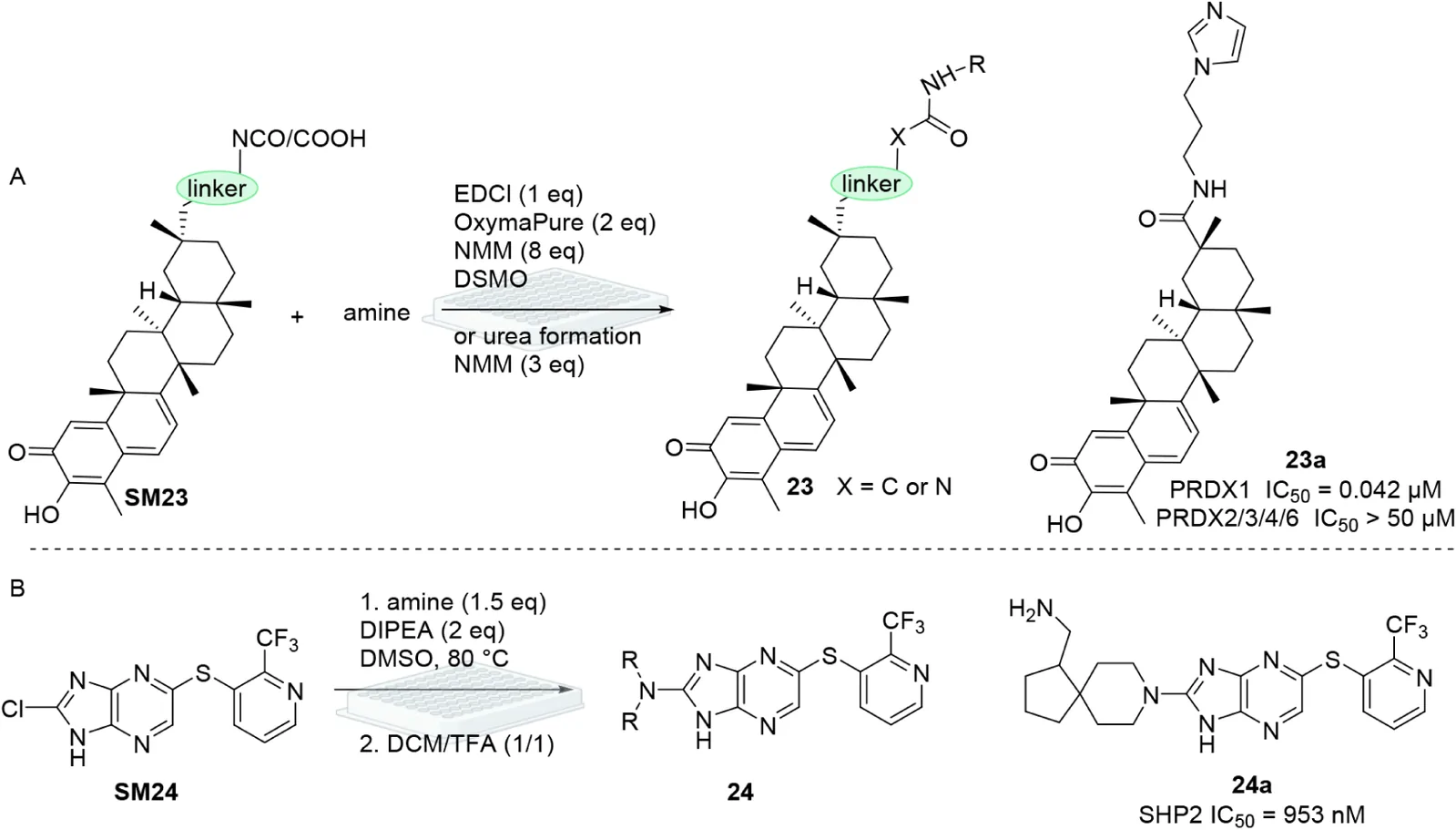
D2B Workflows Employing Coupling Reactions[Fn sch6-fn6]

a (A) Parallel amide coupling and Curtius rearrangement to generate PRDX1 inhibitor libraries. (B) Plate-based amide coupling with subsequent Boc deprotection for the discovery of SHP2 inhibitors.

Ponzi and coworkers reported a D2B campaign targeting allosteric Src homology region 2 tyrosine phosphatase 2 (SHP2) inhibitors (Scheme 6B).^35^ Guided by cocrystal structural analysis, a fused tricyclic amine scaffold **SM24** was diversified via nucleophilic aromatic substitution followed by Boc deprotection in 96-well plates (80 °C, DMSO/DIPEA). In total, 160 structurally diverse amines were coupled at ∼3 mg scale per well. After solvent removal (Lyospeed), crude products **24** were diluted and evaluated directly in a fluorescence-based 6,8-difluoro-4-methylumbelliferyl phosphate phosphatase assay. QC analysis identified 133 reactions exceeding 30% purity, which were screened at four concentrations; 29 compounds showed >50% inhibition and four reaction mixtures exhibited nanomolar activity. The resynthesized hits confirmed activity, and stereochemical analysis revealed a 4-fold potency advantage for the trans isomer of the lead compound.

Kitamura, Wolan, and coworkers developed a sulfur–fluoride exchange (SuFEx)–based D2B platform enabling rapid diversification of iminosulfur oxidifluoride warheads with large amine libraries, allowing crude reaction mixtures to be screened directly against purified protein targets. Their initial implementation targeted the bacterial cysteine protease SpeB,^36^ where benzyl­(cyanomethyl)­carbamate scaffolds (**SM26a**) were converted overnight into 460 derivatives (**26**) via SuFEx in 96-well plates (10 nmol scale, DMSO/PBS 1:1, 37 °C, 80 μL) (Scheme 7A). Representative wells were analyzed by Liquid Chromatography–Charged Aerosol Detection–Mass Spectrometry (LC–CAD–MS), revealing moderate-to-high conversion for primary and cyclic secondary amines. Crude mixtures were diluted and screened in a fluorogenic SpeB activity assay, yielding nanomolar inhibitors. Miniaturization to a 1536-well format (400 pmol, 2 μL) using acoustic dispensing preserved activity trends, with product formation again confirmed by LC–MS. A lead compound **26c** of this series demonstrated antibacterial activity in a toxicity assay targeting neutrophils.

**Scheme 7:**
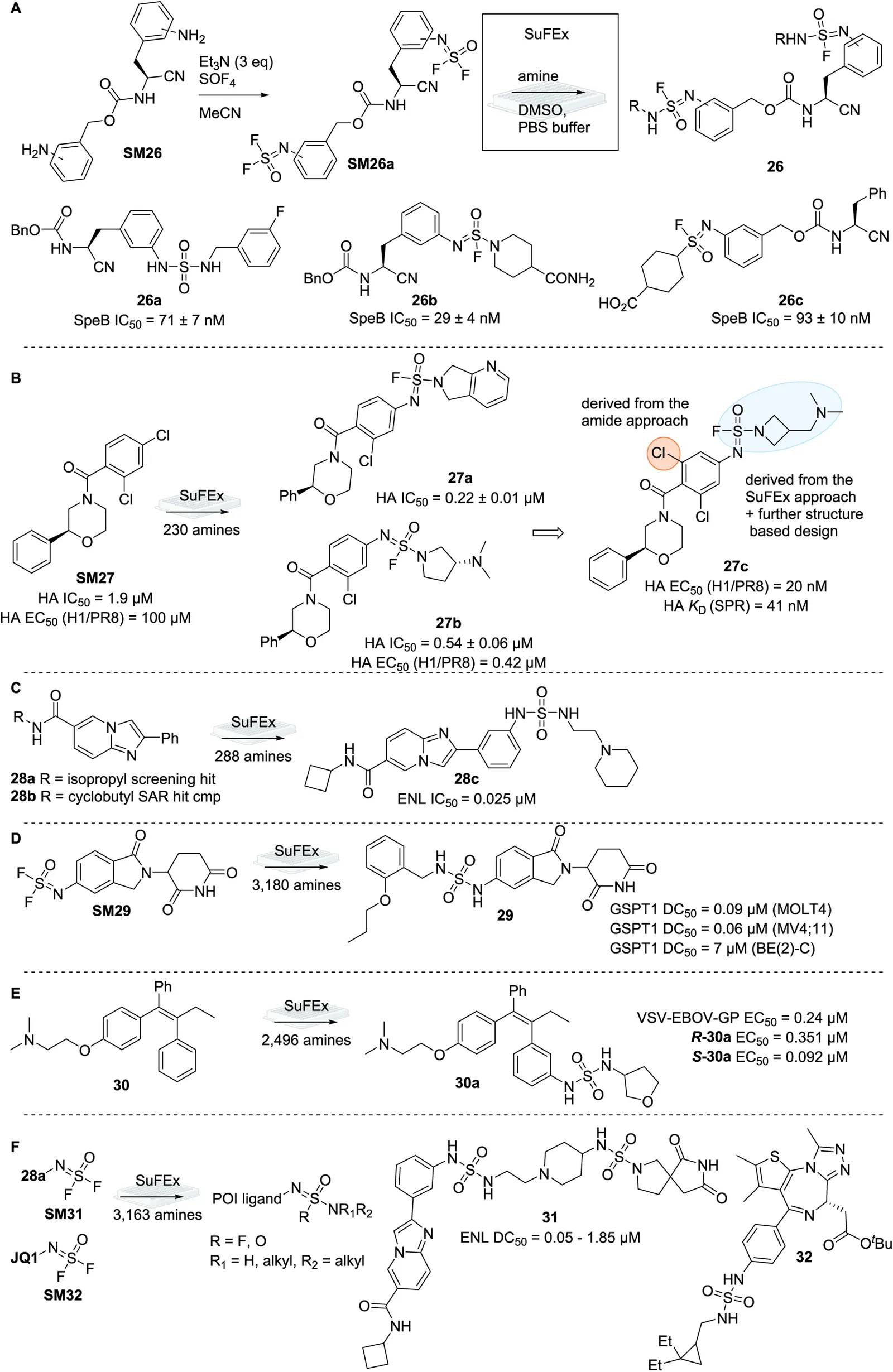
SuFEx Chemistry Implemented in D2B Workflows[Fn sch7-fn7]

a (A) Screening for SpeB inhibitors, (B) to afford inhibitors of influenza hemagglutinin, (C) to identify hits targeting ENL, (D) to screen for molecular glues for GSPT-1, (E) to screen for Ebola virus entry inhibitors, and (F) screening for chemical inducers of proximity for ENL and BRD4.

To demonstrate the broader applicability of the platform for the development of antiviral agents, it was used to target influenza hemagglutinin (HA) (Scheme 7B).^37^ Structure-guided installation of iminosulfur oxydifluoride handles enabled diversification with 230 amines in 384-well plates, alongside a parallel amide-coupling library (∼500 carboxylic acids, HBTU 1.1 equiv, DIEA 2 equiv, DMF, RT, 50 μL). Reaction progress was verified by LC–MS on representative wells. Crude products (e.g., **27a** and **27b**) were screened using a fluorescence polarization assay measuring displacement of a TAMRA-labeled peptide from the HA stem pocket. Starting from a hit with a cellular EC_50_ of 100 μM, iterative SuFEx optimization improved potency to a cellular EC_50_ of 0.42 μM. Subsequent integration of SuFEx- and amide coupling-derived SAR delivered compound **27c** with a cellular EC_50_ of 0.02 μM while rapidly eliminating unproductive exit vectors.

The predominance of recombinant protein assays in current D2B workflows highlights an important trade-off between robustness and biological relevance. Their tolerance toward residual reagents and straightforward quantitative readouts makes them ideally suited for high-throughput screening of crude reaction mixtures. However, because they assess isolated protein function, they provide limited insight into cellular activity, permeability, and toxicity. Future D2B platforms will therefore increasingly benefit from integrating robust recombinant assays for primary hit identification with orthogonal cellular assays for downstream validation.

### D2B Using Cellular Assay Readouts

The robustness that established SuFEx as a leading chemistry for recombinant D2B assays also enabled its successful translation to cellular screening formats. For the chromatin reader 11–19 leukemia (ENL) in leukemia,^38^ assay compatibility was first confirmed by fluoride-spiking controls in a homogeneous time-resolved fluorescence (HTRF) assay. An iminosulfur oxydifluoride handle was installed on initial hits (**28a, 28b**) and reacted in parallel with a 288-member amine set, with crude products screened directly in an ENL YEATS domain HTRF assay (Scheme 7C). Although biochemical potency improved substantially, these activity gains did not translate to *K*_D_s measured in cellular target engagement assays. To address this disconnect, an optimized SuFEx hub was diversified with histamine-like amines and crude products were screened directly in living cells using an ENL­(YEATS)-HiBiT assay, revealing a preference for piperidine substituents and enabling identification of **28c** despite it not being the most potent analogue in HTRF.

Building on this cellular D2B capability, the approach was extended to molecular glue discovery targeting cereblon (CRBN).^39^ 5-Amino-lenalidomide was converted to an iminosulfur oxydifluoride moiety (**SM29**) and reacted with primary and secondary amines in 384-well plates (10 μL 1:1 DMSO:PBS, 2 mM, 37 °C), generating 3,180 crude derivatives, with representative wells analyzed by UPLC–MS (Scheme 7D). Phenotypic screening in MOLT4, MV4;11, and BE(2)-C cells using luminescence-based viability assays identified 19 hits (e.g., **29**) inducing CRBN-dependent antiproliferative effects. Follow-up studies in CRBN-depleted and G1 to S phase transition protein 1 (GSPT1) mutant cells established GSPT1 degradation as the dominant mechanism, underscoring the importance of orthogonal assays in phenotypic SuFEx screening.

Most recently, the platform was applied to Ebola virus entry inhibitors (Scheme 7E).^40^ A tamoxifen (**30**)-derived SuFEx handle was reacted with 2,496 amines in 384-well plates (1 mM, DMSO/phosphate buffer, overnight), with representative conversions confirmed by LC–MS. After a 5000-fold dilution, crude products were screened at 200 nM in a virus-like particle assay using vesicular stomatitis virus expressing Ebola glycoprotein to infect Vero cells, with infection quantified by eGFP fluorescence normalized to stained nuclei. Resynthesized hits showed nanomolar potency, with the best enantiomer (***S*** **-30a**) achieving EC_50_ = 92 nM.

Leggott and coworkers reported an activity-directed D2B workflow for antibacterial discovery that couples Pd-catalyzed microscale synthesis with direct phenotypic screening against *Staphylococcus aureus* (Scheme 8A).^41^ Quinazolinone libraries were generated via Pd-catalyzed carbonylation/amination cascades from *o*-iodoanilides (**33**) and diverse amines **SM34** in 96-well plates (333 mM limiting substrate in *o*-xylene) using Pd_2_(dba)_3_ (0.1 mol %), P­(*t*Bu_3_)­HBF_4_ (0.6 mol %), Mo­(CO)_6_ (1 equiv), and Et_3_N at 105 °C for 48 h, without stirring or inert atmosphere. In total, 220 reactions were executed. Crude mixtures **34** were filtered, evaporated, redissolved in DMSO, and screened directly at 50 μM against three *S. aureus* strains following assay optimization for solubility. Six reproducibly active wells were scaled up and purified by mass-directed HPLC, affording 2,3-disubstituted quinazolinones (**57**) with MIC values of 0.016–4 μg mL^–1^, including lead **34a** (38 nM), which showed ∼10-fold improved potency over the reference and activity against MRSA USA300 JE2. SAR analysis revealed strict requirements for meta-substituted aryl groups and hydrophobic C2 substituents, with inactive crude reactions translating to weak purified compounds, underscoring the fidelity of crude-to-pure ranking.

**Scheme 8:**
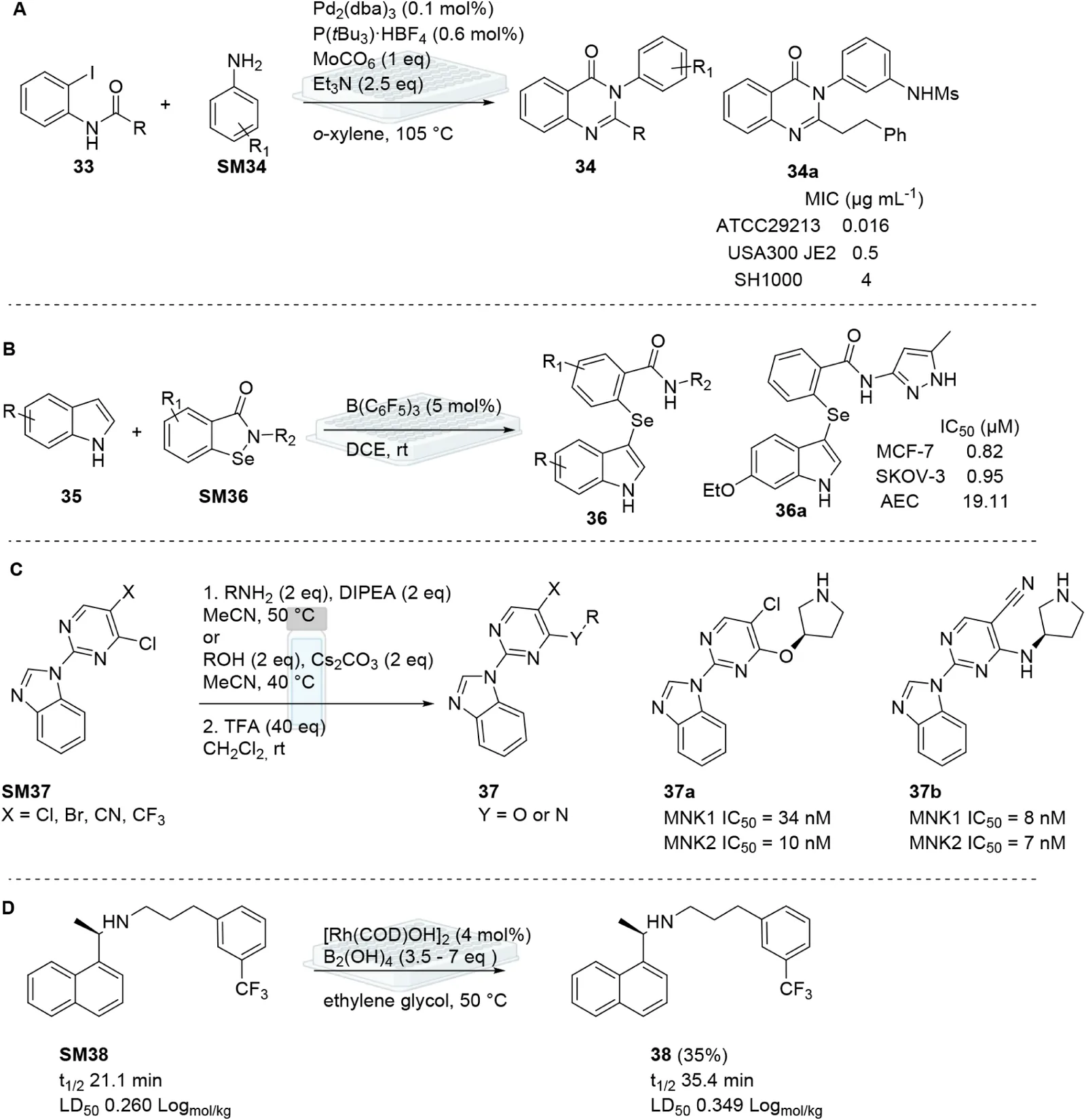
Diverse D2B Workflows Employing Advanced Biological Readouts[Fn sch8-fn8]

a (A) Pd-catalyzed cross-coupling for antibacterial hit discovery. (B) C3–H selenylation for antiproliferative hit discovery. (C) S_N_Ar chemistry for the discovery of MNK inhibitors. (D) Rh-catalyzed late-stage saturation followed by direct microsomal stability assessment.

Xu, Wang, and coworkers developed a B­(C_6_F_5_)_3_-catalyzed C3–H selenylation of indoles specifically designed for on-plate parallel synthesis and direct biological screening, extending D2B chemistry beyond conventional medicinal chemistry transformations.^42^ The metal-free reaction between indoles **35** and benzoselenazolones **SM36** proceeded under mild conditions (5 mol % B­(C_6_F_5_)_3_, DCE, room temperature, 12 h) with broad functional-group tolerance and high robustness across solvents, temperatures, and reaction scales (Scheme 8B). Parallel synthesis was performed in 96-well microplates by combining 50 indoles and 27 benzoselenazolones, generating a 1350-member library on a 10 μmol scale in 100 μL reaction volumes without purification. LC–MS analysis showed that 61% of reactions achieved >90% conversion, 83% exceeded 80% conversion, and 91% reached >70% conversion. The crude library **36** was screened in situ for antiproliferative activity in MCF-7 and SKOV-3 cancer cells, leading to identification of compound **36a** with nanomolar antiproliferative activity while exhibiting substantially lower toxicity toward primary normal airway epithelial and fibroblast cells. The chemistry was further translated to DNA-encoded library (DEL) synthesis, confirming its DNA compatibility and the utility of C3–H selenylation as a robust D2B transformation.

Starting from the cocrystal structure of the MNK inhibitor NUCC-0201049 bound to MNK2, Vagadia and coworkers developed a D2B workflow for rapid kinase inhibitor optimization.^43^ Five chloropyrimidine intermediates **SM37** derived from the crystallographic lead were dispensed into 1 mL glass vials arranged in a 96-well reaction block and diversified by S_N_Ar chemistry (Scheme 8C). To streamline library production, stock solutions of the intermediates in dichloromethane were distributed into the reaction block and the solvent was removed by centrifugal evaporation prior to synthesis. Each intermediate was reacted with 16 amine building blocks (2 equiv) and Hünig’s base (2 equiv) in acetonitrile at 50 °C for 16 h, generating an 80-member library. Following solvent removal, Boc deprotection was performed using TFA in dichloromethane at room temperature for 16 h, and residual solvent and acid were removed under nitrogen. Workflow optimization revealed that complete removal of TFA was essential for reliable acoustic liquid handling and biological testing. Crude-to-purified benchmarking demonstrated excellent agreement between activities measured from reaction mixtures and purified compounds, validating direct screening. The amine-based S_N_Ar reactions generally proceeded with higher conversion and cleaner product profiles than the corresponding alcohol-derived analogues. For the alcohol series, substitution initially required the strong phosphazene base BTPP, but crude reaction mixtures displayed reduced apparent potency and flatter dose–response curves than the corresponding purified compounds, highlighting the potential impact of reagent carryover on D2B assay performance. To address this issue, the reaction conditions were reoptimized using Cs_2_CO_3_ as a milder base, improving compatibility between synthesis and biological screening. The platform was subsequently expanded to amine- and alcohol-derived libraries comprising more than 150 analogues, which were screened directly in LN229 glioblastoma cells using an in-cell Western assay measuring inhibition of eIF4E phosphorylation. This workflow enabled rapid generation of cellular SAR and identification of nanomolar MNK inhibitors (e.g., **37a**, **37b**) without purification of the majority of synthesized compounds, illustrating how conventional medicinal chemistry transformations can be successfully integrated into D2B campaigns when reaction performance, reagent compatibility, solvent handling, and assay tolerance are carefully optimized.

Beyond target-based biochemical and cellular activity assays, D2B workflows have also been applied to the direct evaluation of developability properties. To demonstrate compatibility with D2B workflows, Liu, Cernak, Glorius, and coworkers translated their Rh-catalyzed late-stage saturation reaction to an ultrahigh-throughput format.^44^ Aromatic drug molecules (e.g., **SM38**) were hydrogenated using [Rh­(COD)­OH]_2_ (4 mol %) and B_2_(OH)_4_ (3.5 or 7.0 equiv) in anhydrous ethylene glycol at 50 °C for 24 h (Scheme 8D). The reaction was performed on a miniaturized ∼50 μg scale in 1536-well plates, enabling saturation of a 768-compound drug library, with product formation observed for 690 compounds (42.9% average conversion). However, implementation required an extensive sample-preparation workflow, including UPLC–MS analysis of the starting library, robotic transfer from 384- to 1536-well plates, repeated solvent evaporation and ethanol washes to completely remove DMSO, reconstitution in ethylene glycol, multiple dispensing and mixing steps, and postreaction UPLC–MS analysis prior to biological testing. Selected crude reaction mixtures were subsequently transferred directly to a microsomal stability assay, illustrating that while the chemistry is compatible with purification-free screening, substantial upstream handling was required to adapt the compound collection to the reaction conditions.

The principal advantage of cellular D2B lies in its increased biological relevance. By integrating intracellular target engagement, permeability, cytotoxicity, and phenotypic effects into a single workflow, cellular assays provide substantially more informative early-stage data than recombinant protein assays. This additional information comes at the cost of greater sensitivity toward residual catalysts, coupling reagents, solvents, and reaction byproducts, making reaction compatibility considerably more important than for purified protein assays. Consequently, the successful implementation of cellular D2B depends not only on robust synthetic transformations but also on careful optimization of reagent carryover, solvent handling, and assay tolerance.

### D2B Platform for Degrader Development

The development of degraders, such as PROTACs, usually requires extensive optimization of the linker moieties that tether the “protein of interest ligand” (POI) to an E3 ligase. Additionally, the most suitable E3 ligase for efficiently degrading a target is unknown. Consequently, many different E3 ligand systems with diverse linker attachment points must be tested. Similarly, molecular glues require extensive modification of the solvent-exposed moiety to achieve selective target degradation. These properties make small-molecule degraders ideal candidates for a D2B workflow. Cellular systems that detect POI levels are typically used, comprising HiBiT-tagged POI systems or POI fusion with a fluorescent protein.^45–47^ In the latter case, care must be taken to ensure that the detected degradation is mediated by ubiquitination of the POI rather than the fluorescent protein. Therefore, proof of endogenous POI degradation will be required after a D2B screening campaign (Figure 2).

**Figure 2:**
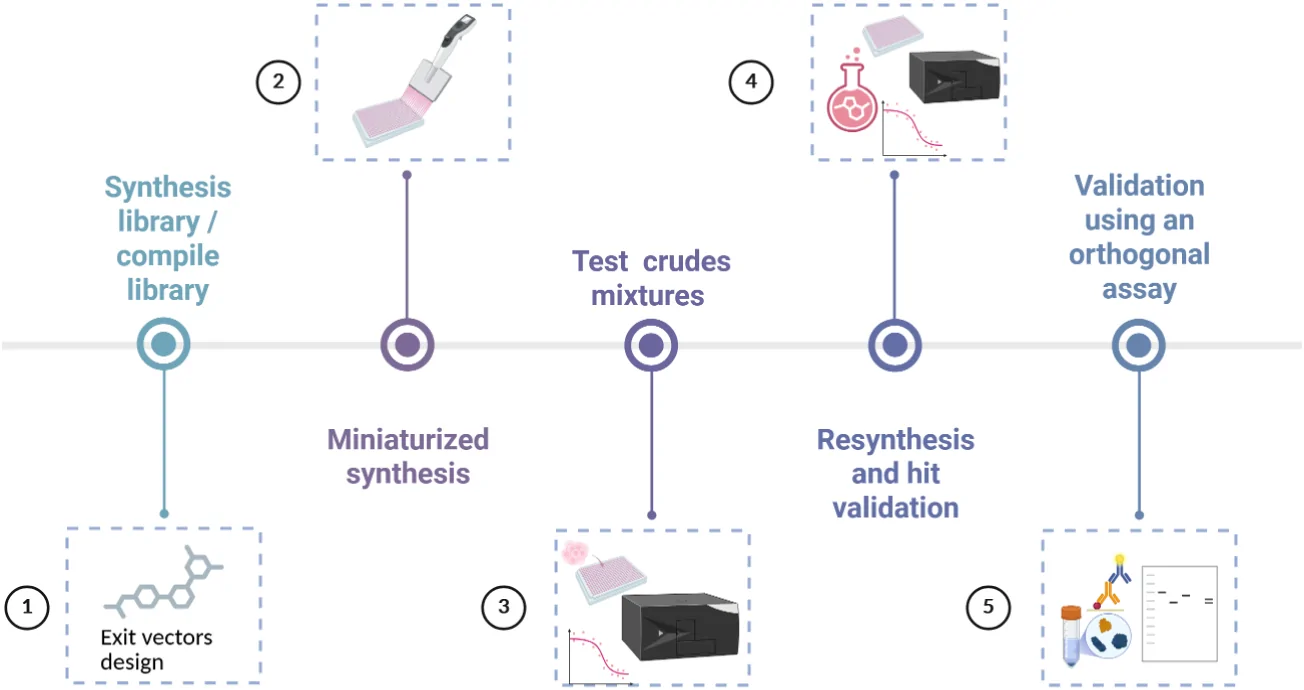
General D2B workflow for degrader discovery. (1) Design of a degrader library by variation of exit vectors, linker attachment points, or warheads. (2) Miniaturized automated synthesis of the library. (3) Direct screening of crude reaction mixtures in a degradation assay. (4) Resynthesis and validation of prioritized degraders. (5) Orthogonal biological and biochemical characterization of validated hits.

Roberts, Ma, and coworkers reported three complementary D2B platforms for PROTAC discovery based on high-yielding ligation chemistries (Scheme 9A). In their initial study, hydrazides **SM40** were condensed with aldehyde-functionalized POI ligands (**39**) to generate acyl-hydrazone–linked PROTACs targeting the estrogen receptor (ER).^48^ Reactions were performed at 1 μmol scale in DMSO (75 °C, 30 h), yielding ∼100 crude PROTACs with LC–MS conversions of typically >90%, although two exit vectors resulted in reduced efficiency (70–80%). Crudes were screened in MCF-7 cells by in-cell ELISA, identifying only von Hippel-Lindau (VHL)-recruiting degraders as active. Active PROTACs with short linkers were prioritized, with **40a** showing DC_50_ = 7.7 nM and *D*_max_ = 95%. Replacing the acyl-hydrazone with a stable amide (**40b**) degradation activity after resynthesis. The same chemistry was miniaturized to 96-well plates (1 μmol, 20 μL) to generate 1,520 GSPT1-targeting molecular glues **42**, which were screened directly in RS4;11 and MOLT4 cells at 1 μM (Scheme 9B).^49^ Lead compounds (**42a**, **42c**) were validated by dose–response, Western blotting, and proteomics. Subsequent replacement of the hydrazone linker with amides yielded analogues **42b** and **42d** (DC_50_ ≈ 0.17 μM) with activity comparable to the original hits, again demonstrating that bioisosteric replacement of an initial high-yielding linkage can retain cellular degradation potency.

**Scheme 9:**
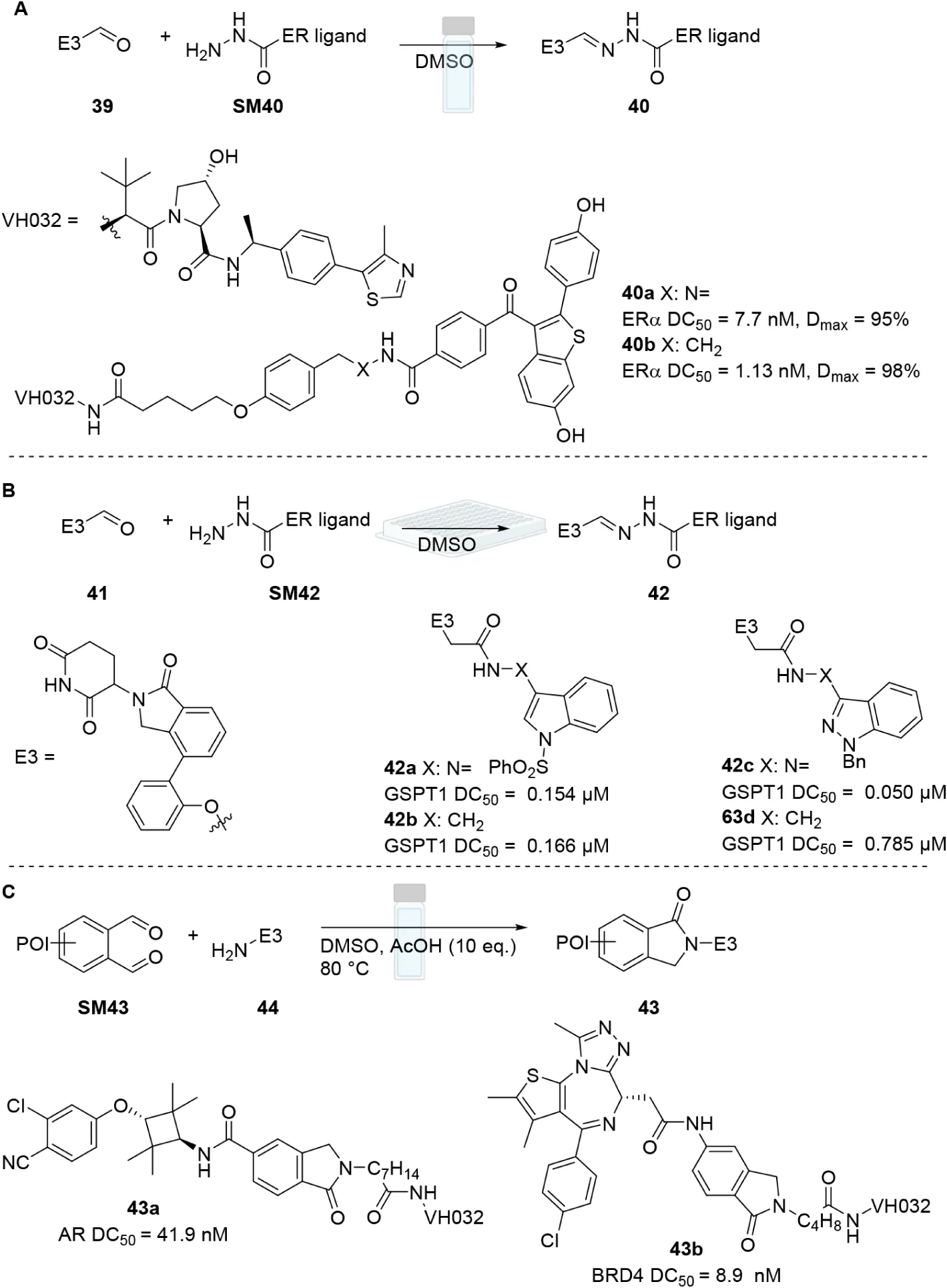
D2B Workflows Employing Condensation Reactions for Targeted Protein Degradation[Fn sch9-fn9]

a (A) Acyl hydrazone-based PROTAC library screened by in-cell ELISA. (B) Acyl hydrazone-based molecular glue library targeting GSPT1 screened in a phenotypic cell assay. (C) Lactam-based PROTAC library screened by Western blot for BRD4 and androgen receptor (AR) degradation.

In a third platform, Guo et al. introduced an *ortho*-phthalaldehyde (OPA) ligation strategy, forming lactam-linked PROTACs from OPA-functionalized POI ligands (**SM43**) and amine-bearing E3 ligase ligands **44** (Scheme 9C).^50^ Optimization showed that DMSO with 10 equiv of acetic acid gave ∼99% model conversion. OPA-modified androgen receptor (AR) ligands were reacted with 21 amines at 80 °C for 48 h, affording crude products with 88–98% HPLC purity. Western blot screening in LNCaP cells identified VHL-based degraders (most active: **43a**, DC_50_ = 41.9 nM). Regioisomer separation revealed no major activity differences. Extension to BRD4 using OPA-functionalized JQ1 gave 78–96% conversion and nanomolar degraders in MV-4–11 cells, with one regioisomer reaching DC_50_ = 8.9 nM (**43b**).

In 2022, linker effects on PROTAC performance were explored using the BRD4 degrader dBET1 (JQ1–alkyl–pomalidomide) as a model.^51^ Diamine linkers (**SM45**) were coupled to NHS-activated JQ1 and CRBN ligands using DIPEA, followed by a telescoped plate-based three-step workflow at a 10 μmol scale in a 50 μL volume (amide coupling, resin scavenging, Boc deprotection, second coupling) (Scheme 10A). Guided by structure-based clustering, 91 PROTACs (**45**) were prepared in parallel with pomalidomide or tolyldihydrouracil (tDHU) CRBN ligands. Conversions were quantified by CAD, giving average yields of 27–32%, and CAD calibration enabled accurate concentration assignment of crudes. The mixtures were evaluated by NanoBRET (live/permeabilized HEK293), HiBiT-BRD4 degradation, and CellTiter-Glo assays. Crude and purified activities correlated well, validating CAD-based quantification. Relative binding affinity (NanoBRET ratios) served as a permeability proxy, revealing higher permeability for compounds with fewer H-bond donors/acceptors and greater lipophilicity. Linker SAR was highly context-dependent: flexible and short rigid linkers varied with CRBN chemotype, whereas longer rigid linkers produced more consistent degradation. Overall, the study demonstrated how D2B chemistry can rapidly establish data-rich linker SAR and it highlights the interplay between linker design, permeability, and degradation efficacy.

**Scheme 10:**
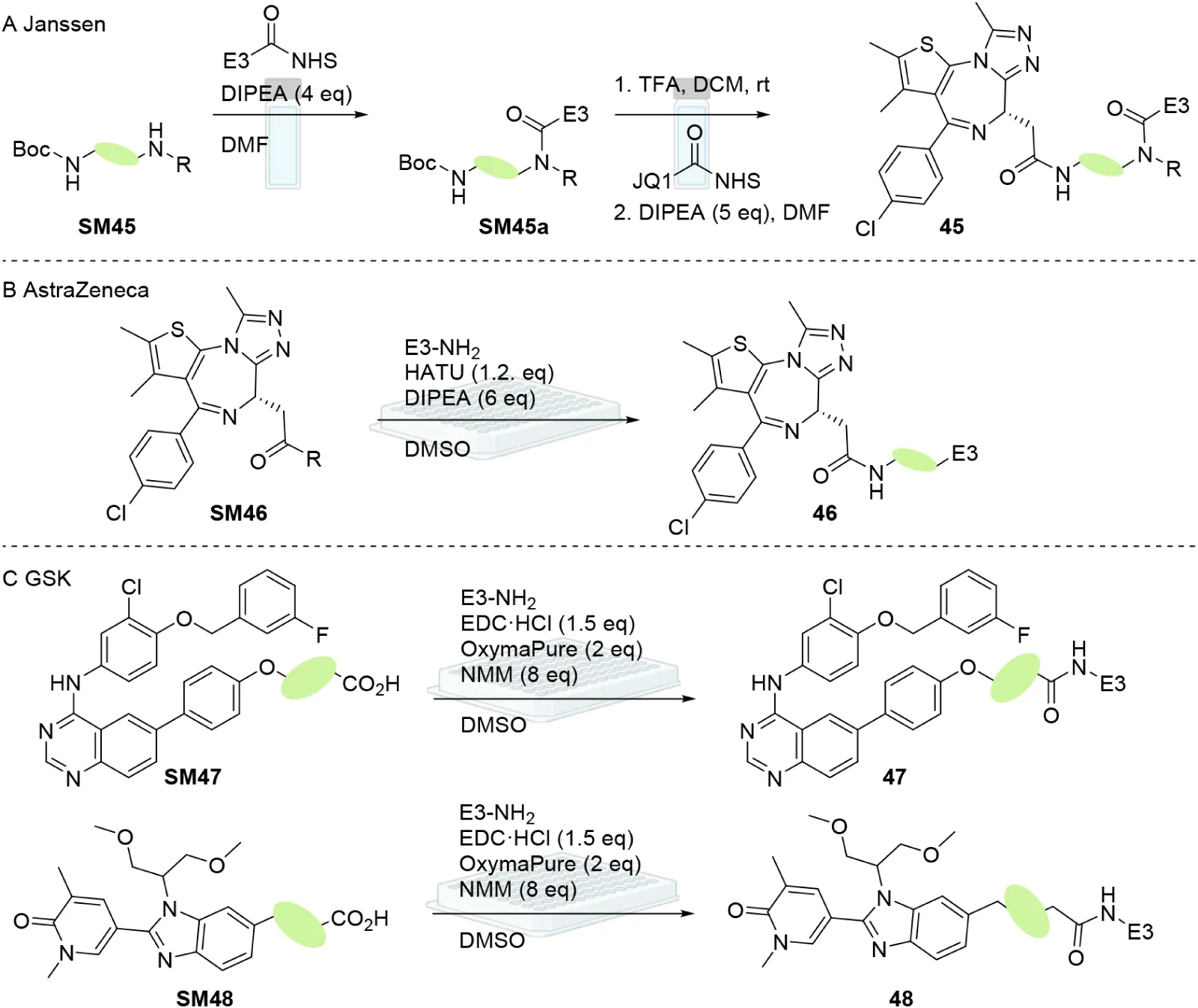
D2B Workflows Employing Amide Coupling for Targeted Protein Degradation[Fn sch10-fn10]

a (A) Janssen's BRD4 PROTAC discovery workflow using HiBiT and NanoBRET assays. (B) AstraZeneca's BRD4 PROTAC discovery workflow using a HiBiT assay. (C) GSK's discovery of HER2- and BRD4-targeting degraders using HiBiT assays.

In 2023, AstraZeneca^52^ and Stevens et al. at GSK^53^ independently reported plate-based D2B platforms for high-throughput PROTAC synthesis via amide coupling. AstraZeneca implemented a 384-well workflow (120 nmol in 20 μL DMSO) using HATU/DIPEA to couple two JQ1-derived BRD4 ligands **SM46** with 34 amine-functionalized E3 ligase linkers (Scheme 10B), ∼50% designed with short, rigid architectures and embedded basic nitrogen atoms. LC–MS showed ∼79% average conversion. Crudes **46** were screened directly in HiBiT degradation and viability assays, yielding picomolar BRD4 degraders with strong crude–pure concordance. Spiking studies caused only modest dose–response shifts, while Western blot and thalidomide cotreatment confirmed target specificity and proteasome dependence. Physicochemical parameters (logD, ePSA) measured from crude samples correlated well with purified compounds.

In parallel, GSK established a 1536-well D2B platform (150 nmol in 5 μL) using a Mosquito robot for dispensing (Scheme 10C). A coupling-reagent screen (EDC, DIC, HATU, TSTU, TCFH, PDCP, COMU) identified HATU and EDC as most effective by LC–MS; EDC was selected due to HATU-associated cytotoxicity. Because DIPEA was immiscible at nanoscale volumes, *N*-methylmorpholine (NMM) was adopted, and OxymaPure suppressed *N*-acylurea formation. Under these conditions (1.5 equiv acid, 1.5 equiv EDC·HCl, 2 equiv OxymaPure, 8 equiv NMM), 80–100% conversion was achieved across most amines (meta-amide anilines ∼30%). Validation of this setup was carried out using human epidermal growth factor receptor 2 (HER2) PROTACs as a target, combining two acid-functionalized HER2 ligands **SM47** with 29 VHL or cereblon amines and 0–20 atom linkers. This screen yielded several active HER2 degraders **47**. Two resynthesized hits confirmed the activity of these PROTACs, though the crude samples exhibited stronger hook effects due to residual starting material competing with PROTAC binding. Additionally, a PROTAC optimization campaign targeting BRD4 using derivatives of I-BET469 as a ligand (**SM48, SM49**) yielded 818 PROTACs (**48**) in two setups. Purified hits showed proteasome-dependent degradation and strong agreement with the degradation data measured on crude products. The second linker-focused library delivered nano- to picomolar degraders, revealing cereblon preference and an optimal linker length of ∼12–15 atoms, with both flexible and semirigid linkers. Collectively, these studies demonstrate that amide-coupling D2B platforms can be rigorously optimized for nanoscale automation, reagent compatibility, and cellular assay tolerance, enabling rapid degrader discovery, direct screening of crude reaction mixtures, and early integration of biological and physicochemical profiling in PROTAC optimization. In a follow-up study, Stevens and coworkers optimized and validated multiple reaction classes (reductive amination, S_N_Ar, alkylation, and Pd-mediated cross-coupling) for D2B PROTAC synthesis (Scheme 11).^54^ Most of the D2B reactions were performed in a glovebox with a Mosquito robot under nitrogen atmosphere. For reductive amination, eight amine-functionalized BRD4 ligands (**SM49**) were reacted with three aldehyde-bearing E3 ligase ligands (**SM49a**). Sodium triacetoxyborohydride resulted in poor conversion and significant side products, whereas picoline borane achieved full conversion in 13 of 24 cases, yielding 50–80% crude purity. Crudes (**49**) were directly screened in a HiBiT assay, identifying potent BRD4 degraders with strong correlation with seven resynthesized purified compounds. Interestingly, a 10-fold excess of picoline borane did not affect assay performance. For the optimization of S_N_Ar reactions, soluble organic superbases were screened, and MTBD was selected because of its high conversion and DMSO compatibility. Six heteroaryl E3 ligase ligands (pyrazine, pyrimidine, pyridazine) were reacted with eight BRD4 ligands containing an amine at 60 °C in 5 μL DMSO using a mosquito liquid handler, achieving quantitative LC–MS conversion across all 48 reactions. Three compounds were resynthesized, one of which showed poor correlation with the corresponding crude. However, base spiking was tested as a factor influencing assay performance but showed no interference. Additionally, a multistep Pd-mediated C­(sp^2^)–C­(sp^3^) Suzuki–Miyaura workflow was developed, combining cross-coupling in vials with Boc deprotection and plate-based amide coupling. Using RuPhosPd G3 (30 mol %), K_3_PO_4_, toluene/water at 100 °C, the coupling product was the major LC–MS species. Four cleanup strategies (HPLC, Celite, SPE, aqueous workup) were evaluated prior to TFA deprotection, SCX purification, and final amide coupling. Resulting BRD4 PROTACs (**50**) showed only 20–40% purity, with *D*_max_ more sensitive to low conversion rates than DC_50_ values. This example highlights the breadth of what is currently described as D2B and illustrates that, in practice, such workflows can approach parallel synthesis, particularly when intermediate purification steps are required. While crude testing is still retained in the final step, the need for partial cleanup underscores the continuum between classical parallel synthesis and fully integrated D2B approaches.

**Scheme 11:**
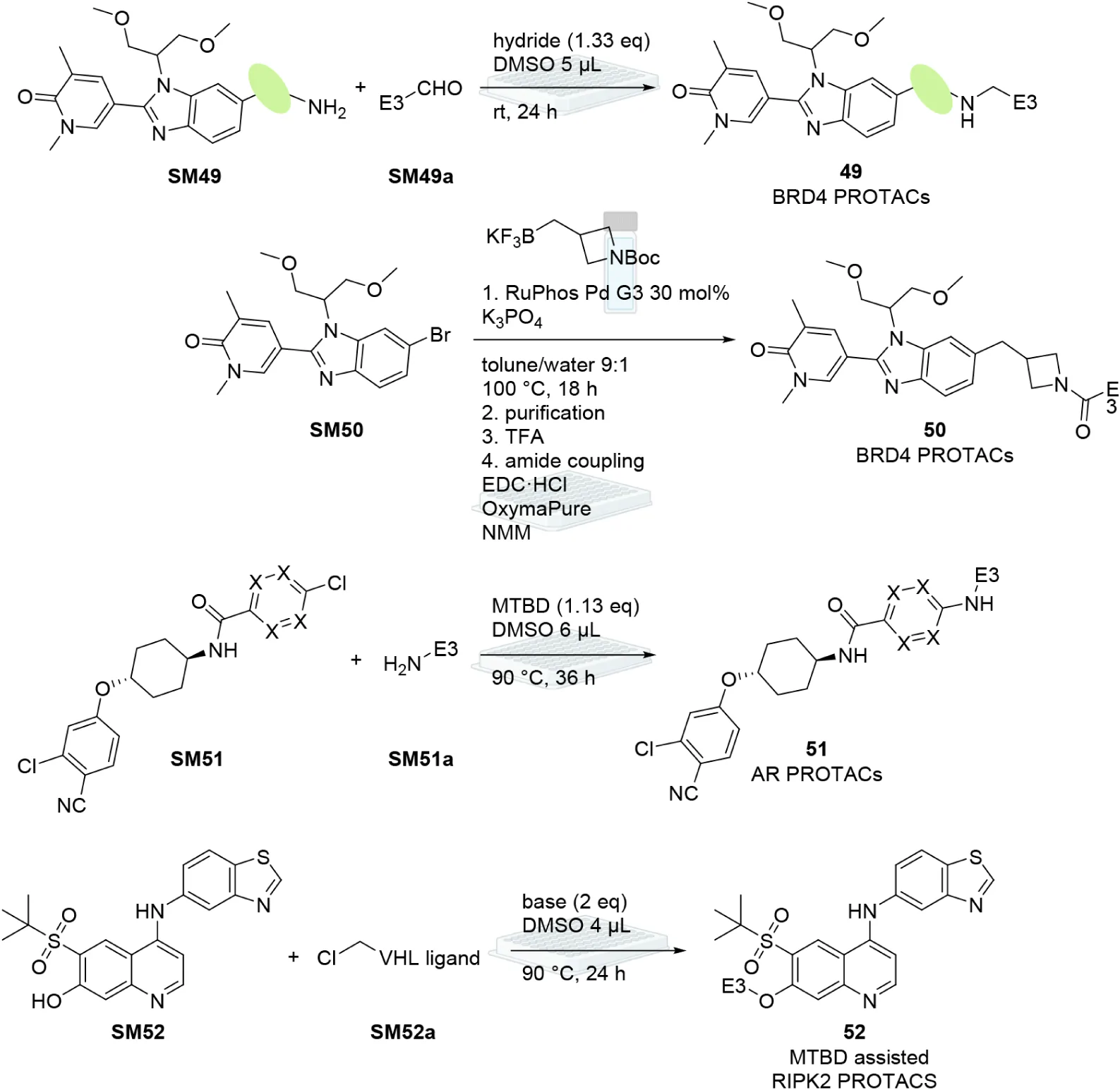
Representative Reaction Classes Implemented in GSK D2B Workflows: Reductive Amination, Pd-Catalyzed Alkylation Followed by Amide Coupling, S_N_Ar Reactions, and Alkylation

S_N_Ar chemistry was further applied to androgen receptor (AR) degraders inspired by ARV-110, coupling AR ligands bearing heteroaryl chlorides (**SM51**) to 76 amine-containing E3 ligase ligands (**SM51a**) using MTBD at 90 °C for 36 h. Conversion rates of 74–95% were achieved (65/76 successful), with excellent crude to purified correlation (**51**) and no assay interference detected upon base spiking. Finally, alkylation chemistry was established for receptor-interacting serine/threonine-protein kinase 2 (RIPK2) degraders using a phenol-containing RIPK2 ligand (**SM52**) and alkyl chloride–functionalized VHL ligands **SM52a**. P2-Et favored *N*-alkylation, whereas MTBD promoted *O*-alkylation. Thus, the observed regioselectivity effectively doubled the diversity of the library. The O-alkylated products (**52**) demonstrated superior potency and a strong correlation between the assay data of the crude and purified products. Additionally, no impact from base carryover was observed. Overall, this work demonstrated that D2B chemistry can be systematically diversified. When reaction conditions are finely tuned, the resulting crude products are suitable for reliable biological screening across multiple medicinal chemistry transformations.

Anderson and coworkers at AstraZeneca used the above-reported D2B approaches for the discovery of the first 2′-deoxynucleoside 5′-monophosphate *N*-glycosidase (DNPH1) PROTACs (Scheme 12).^55^ An initial triazole-based DNPH1 ligand (**53**) which was identified by a DNA-encoded library screen and validated by X-ray crystallography, was equipped with a solvent-exposed carboxylic acid exit vector (**SM54**). A 384-well plate amide-coupling workflow was implemented at the 120 nmol scale, using HATU and DIPEA in DMSO to couple the DNPH1 acid with 238 amine-terminated E3 ligase ligands. This series contained diverse E3 ligase chemotypes and linker architectures. LC–MS analysis confirmed product formation in 218 conditions tested, and crude mixtures were screened directly using a HiBiT DNPH1 degradation assay. This strategy identified 28 active degraders (**54**) which included five PROTACs that achieved near-complete DNPH1 depletion (e.g., **54a**). Resynthesized hits showed strong concordance between crude and purified samples, and degradation was confirmed to be VHL- and proteasome-dependent. To improve the potency of the developed PROTACs, a second D2B campaign was initiated that employed a quinazoline-based DNPH1 ligand bearing an aldehyde moiety (**SM55**), enabling reductive amination as an orthogonal D2B-compatible coupling reaction. Reactions were performed in 384-well plates by combining E3 ligase amines (1.2 equiv) with the aldehyde ligand (1.0 equiv), Et_3_N (4 equiv), acetic acid (35 equiv), and 2-picoline borane (2 equiv) in DMSO (10 μL, 20 mM), followed by incubation at 40 °C for 39 h. This generated 377 PROTACs (**55**) with an average conversion rate of ∼53%. Despite the presence of frequent side products of the aldehyde reduction, such as hydroxymethyl quinazoline and variable conversion rates, the crude samples were screened directly. This approach resulted in the identification of nanomolar degraders, including **55a**, which showed only 7% crude conversion yet translated to DC_50_ values comparable to the purified compound. **55a** achieved 100% DNPH1 degradation, sustained knockdown over multiple days, and phenotypic recapitulation of DNPH1 genetic loss, including selective sensitization of BRCA1 mutant cells to 5-hydroxymethyl-deoxyuridine.

**Scheme 12:**
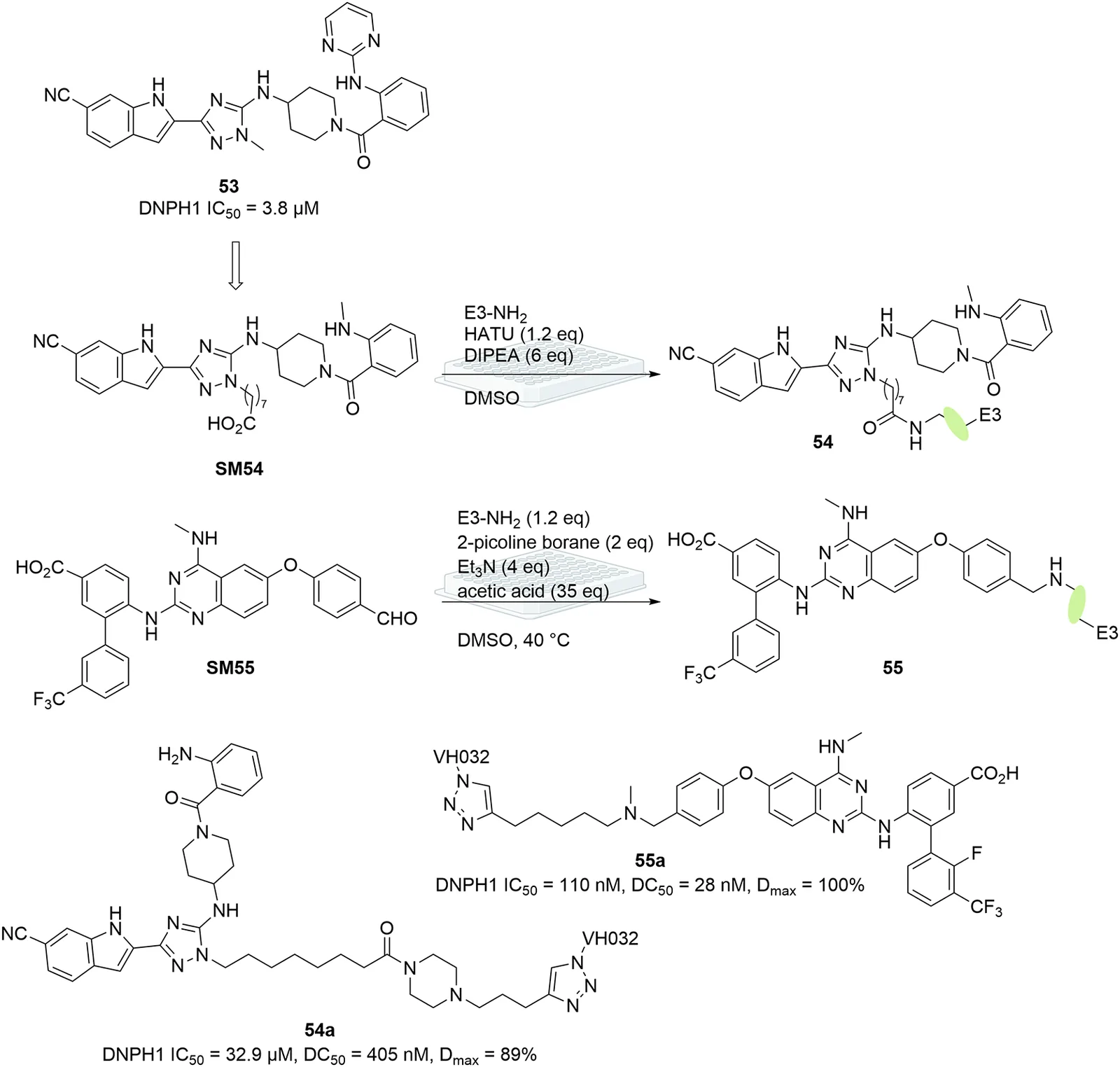
Application of Established D2B Workflows to DNPH1-Targeting PROTAC Discovery at AstraZeneca.

Multicomponent chemistry has recently emerged as a valuable addition to the D2B reaction toolbox. Gao and coworkers first demonstrated its applicability using the Groebcke–Blackburn–Bienaymé reaction to generate a 1,536-member library for menin ligand discovery.^56^ Direct screening identified three validated micromolar binders (best *K*_D_ = 2.8 μM), illustrating that complex heterocyclic libraries can be efficiently generated and evaluated in a D2B format. Building on this concept, Wang and coworkers reported a D2B platform for molecular glue discovery based on isocyanide-driven multicomponent reactions (Scheme 13A).^57^ To rapidly diversify pomalidomide-derived cereblon ligand **56**, three Ugi-type reactions were explored: the Ugi four-component reaction (**57**), the Ugi–formaldehyde reaction (**58**) (which showed the highest success rate), and the Ugi–tetrazole reaction (**59**). Reagents were dispensed into 384-well plates using I.DOT nanodispensing with randomized layouts. The reactions were performed in <1 μL trifluoroethanol at room temperature for 24 h. MS analysis detected product formation in 60% of crudes, which were screened directly in a phenotypic viability assay using MM.1S multiple myeloma cells, with 73% of samples reducing viability below 50%. From these hits, 19 compounds were resynthesized and purified, largely retaining activity. Dose–response profiling identified **57a** and **58a** as more potent ligands compared to pomalidomide in MM.1S and NCI-H929 cells. Mechanistic studies confirmed CRBN-dependent, proteasome-mediated degradation, with **58a** inducing enhanced degradation of IKZF1/3, GSPT1, and the rarely observed target GSPT2: The degradation was confirmed by proteomic analysis. Consistent with its broader degradation profile, **58a** exhibited enhanced and more widespread antiproliferative activity across a diverse cancer cell-line panel.

**Scheme 13:**
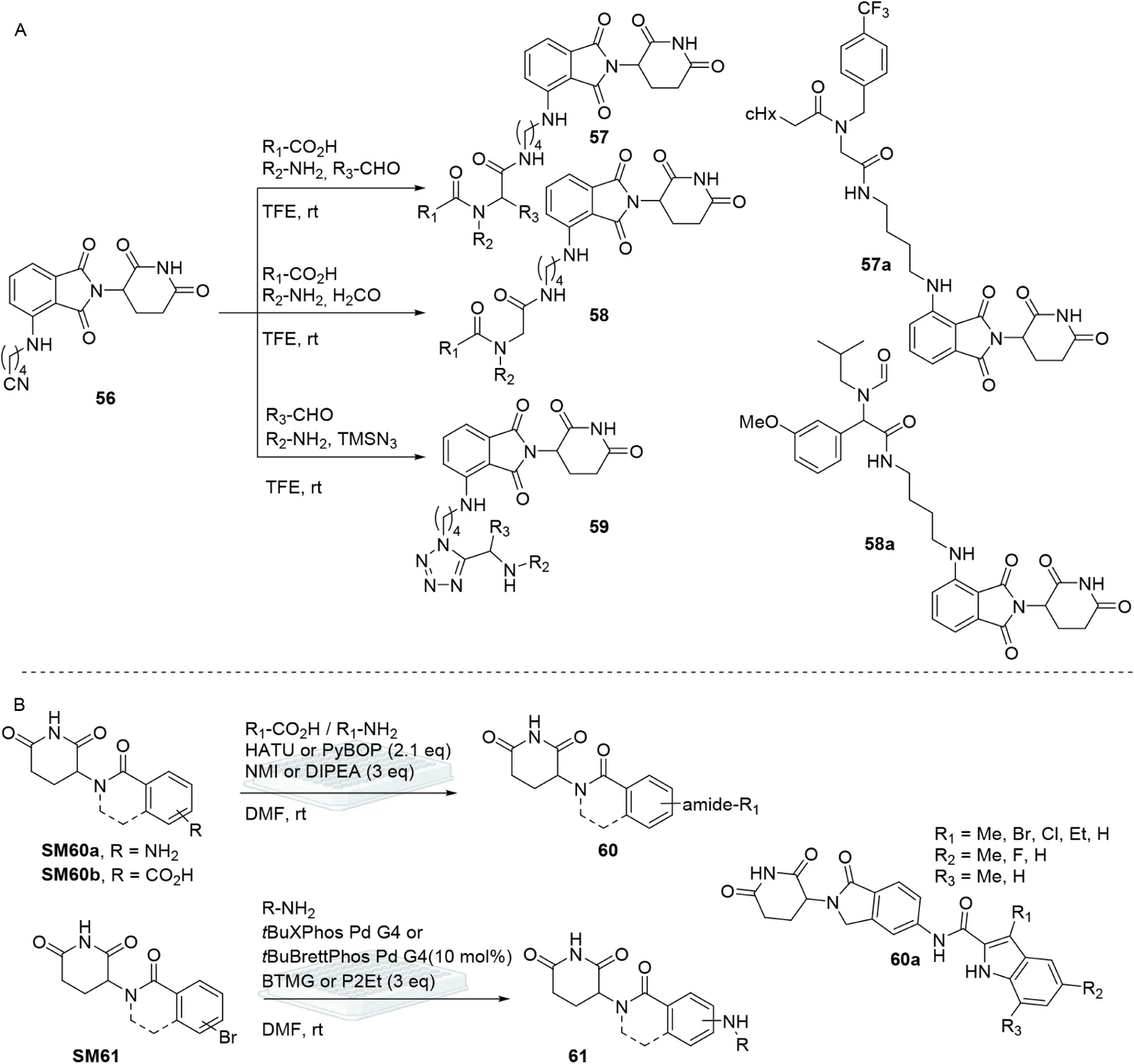
D2B Workflows for Molecular Glue Discovery[Fn sch13-fn11]

a (A) MCR-derived molecular glue library screened by phenotypic assays. (B) Nanoscale high-throughput synthesis coupled with ASMS screening.

Recently, Hu and colleagues reported a D2B molecular glue discovery platform that combined nanoscale high-throughput synthesis with screening by ASMS (Scheme 13B). Goal of the study was to identify molecular glues directly from crude reaction mixtures.^58^ Chemical diversity was generated from 16 cereblon-binding building blocks containing the glutarimide scaffold and distinct exit vectors compatible with amide coupling (**SM60a, SM60b**) and Buchwald–Hartwig amination (**SM61**). Using automated liquid handling, libraries of amines and carboxylic acids were combined in 4 μL reactions under four reaction conditions per scaffold, resulting in >20,000 crude reactions (**60**, **61**). Reaction outcomes were rapidly evaluated by acoustic droplet ejection MS/MS, exploiting diagnostic neutral-loss fragments to identify productive conditions on a per-compound basis. Successfully formed products (4,435 compounds) were algorithmically pooled into 70 mass-resolved mixtures (60 compounds per pool, ≥0.050 Da separation), eliminating the need for individual purification or deconvolution. These crude pools were screened by SEC-coupled ASMS against the CRBN–lymphocyte-specific protein tyrosine kinase (LCK) protein pair, with CRBN-only and LCK-only controls to identify ligands selectively enriched through ternary complex formation. This workflow yielded indole-based molecular glue candidates (**60a**), which were resynthesized and validated by AlphaScreen proximity assays and cellular HiBiT degradation assays, demonstrating strong agreement between ASMS enrichment, ternary complex stability, and cellular activity. Competition experiments showed that potent glues remained enriched in the presence of excess weaker binders, supporting a thermodynamic sink model in which slowly dissociating ternary complexes are preferentially selected. Overall, this study demonstrates that pooled, crude D2B chemistry coupled with kinetic ASMS selection enables scalable, function-first molecular glue discovery, extending D2B strategies beyond degradation-centric or phenotypic screening approaches.

Several groups have demonstrated that CuAAC is well suited for D2B PROTAC discovery, enabling robust ligation chemistry, high reaction conversions, and direct screening of crude reaction mixtures in both miniaturized plate-based and vial-based automated formats (Scheme 14). Chen and coworkers reported Auto-RapTAC, an automation-assisted D2B platform for rapid PROTAC synthesis and evaluation that combines modular building blocks, biocompatible click chemistry, and ready-for-screening assays.^59^ PROTACs were assembled from alkyne-functionalized protein-of-interest ligands and azide-terminated E3 ligase–linker conjugates recruiting CRBN, VHL, mouse double minute 2 homologue (MDM2), and DDB1- and CUL4-associated factor 16 (DCAF16). Assembly relied on copper-catalyzed azide–alkyne cycloaddition, selected for its robustness and compatibility with direct biological screening. Reactions were performed in individual vials under automated control with a reaction volume of approximately 1000 μL using CuSO_4_/TBTA catalysis and sodium ascorbate in DMSO. Optimization identified 1.3 equiv of the E3 ligand, 60 mol % CuSO_4_/TBTA, 40 °C, and a 3 h reaction time as optimal, routinely affording >70% conversion as determined by in-line HPLC. Crude reaction mixtures were transferred directly into biological assays without purification. The platform was validated using cyclin-dependent kinase 2 (CDK2), cyclin-dependent kinase 12 (CDK12), and BCL6 as POIs, enabling parallel synthesis of 96–160 PROTACs per campaign followed by fluorescence-based cellular degradation assays. In the case of CDK2, most PROTACs induced robust degradation in H1299 cells. The resynthesized hits had DC_50_ values in the 300–450 nM range. The screening data showed that VHL-recruiting PROTACs and alkyl linkers of 9–10 carbons were preferred. Extension to CDK12 and BCL6 yielded nanomolar degraders with strong agreement between crude and purified data and minimal hook effects.

**Scheme 14:**
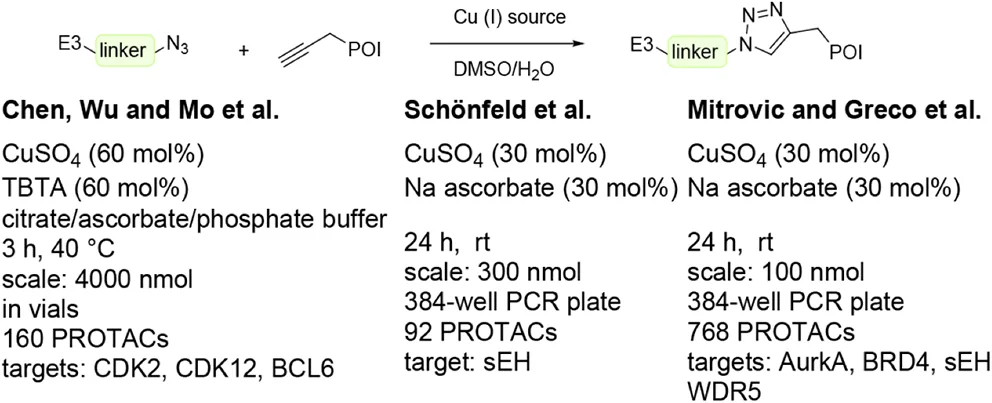
CuAAC Reactions Used in D2B Approaches to Find PROTAC Hits

Schönfeld and coworkers reported a miniaturized D2B platform for PROTAC discovery based on CuAAC using soluble epoxide hydrolase as a model target.^60^ Four alkyne-functionalized soluble epoxide hydrolase (sEH) ligands were combined with 23 azide-terminated CRBN ligands to generate 92 PROTACs. Extensive optimization identified CuSO_4_·5H_2_O/sodium ascorbate as the optimal catalyst system and DMSO/H_2_O (4:1) as a biocompatible solvent. Reaction performance was highly dependent on concentration and plate format, with high conversions only achieved at 30–60 mM reactant concentration and upon transfer from 96-well to 384-well PCR plates. Under optimized conditions (0.3 equiv CuSO_4_·5H_2_O, 0.3 equiv sodium ascorbate, 16 h at room temperature), reactions were miniaturized to 10 μL (300 nmol) while maintaining ∼97–98% conversion. Crude reaction mixtures were screened directly at a concentration of 300 nM using a HiBiT lytic degradation assay. This approach robustly identified potent sEH degraders despite variable conversion rates. Two hits were resynthesized and purified, showing subnanomolar potency and high *D*_max_ (>90%). The degradation of sEH was confirmed to be cereblon- and proteasome-dependent and spiking experiments demonstrated that residual unreacted protein-of-interest ligand reduced degradation through competitive binding. In a follow-up study from Mitrović and colleagues, the workflow was further automated using a Mantis liquid-handling system and reaction scales were reduced further.^61^ PROTACs were assembled from alkyne-functionalized POI ligands and a self-synthesized library of azide-terminated E3 ligase ligands recruiting CRBN and VHL. Reactions were performed in 384-well plates at the 100 nmol scale with reduced reaction volumes of 5 μL in DMSO/H_2_O using CuSO_4_·5H_2_O and sodium ascorbate under mild conditions. Optimization confirmed that reactant concentration, copper loading, and reaction volume governed conversion and assay compatibility, yielding average conversions of ∼80–95% as determined by HPLC–MS. Using this approach, 192 crude PROTACs were synthesized per target by combining four alkyne-functionalized protein-of-interest ligands with 48 presynthesized azide-functionalized E3 ligase–linker conjugates spanning alkyl, PEG, and heterocyclic linker architectures. The platform was validated using the well-established targets BRD4, WD-repeat-containing protein 5 (WDR5), Aurora kinase A (AurkA), and sEH and a HiBiT lytic and NanoLuc live-cell degradation assays. Also in this study, the resynthesized hits showed strong concordance between crude and purified degradation data. Taken together, these studies demonstrate that systematic optimization of CuAAC enables reliable, scalable, and chemically diverse D2B workflows, establishing click chemistry as a robust and flexible alternative to amide coupling for high-throughput PROTAC discovery.

Yan et al. reported a plate-based D2B platform for PROTAC discovery that combines amide coupling with light-induced primary amine-*o*-nitrobenzyl alcohol cyclization (PANAC) photoclick chemistry, enabling seamless synthesis and direct cellular screening of crude reaction mixtures (Scheme 15).^62^ The approach uses *o*-nitrobenzyl alcohol–derived NHS esters (**SM62a**) as modular linkers, allowing flexible assembly of primary-amine-functionalized POI ligands (**SM63**) with E3 ligase ligands (**62**). Reaction conditions were optimized directly in plates. Amide coupling in PBS/DMSO (1:1, pH 10.5) reached near-quantitative conversion within 20 min, followed by PANAC photoclick conjugation upon 365 nm irradiation, affording >95% conversion to **63** within 20 min under metal-free conditions with only *N*-hydroxysuccinimide and water as byproducts. The two-step sequence was compatible with 96-well formats and required no intermediate purification, with basic PBS providing optimal performance. Using this workflow, PROTAC libraries were generated by combining CRBN- and VHL-recruiting E3 ligands and CDK9-derived POI ligands via interchangeable o-NBA-NHS linkers. An initial library of 240 PROTACs (10 E3 ligase ligands × 6 POI ligands × 4 linkers) achieved >70% conversion in 85% of the reactions, and expansion to additional CRBN binders yielded 340 PROTACs with similarly high conversion. Crude products were screened directly in triple-negative breast cancer cell lines (MDA-MB-231 and MDA-MB-468) without assay detected interference. Cell viability screening identified multiple active cyclin-dependent kinase 9 (CDK9) degraders, with purified representatives showing IC_50_ values around 400 nM and strong agreement between crude and purified activity. From the second screen, PROTAC **63a** inhibited MDA-MB-231 cells (IC_50_ = 423 nM) and **63b** inhibited MDA-MB-468 cells (IC_50_ = 269 nM). Western blot and mechanistic studies confirmed CRBN-, proteasome-, and NEDD8-dependent degradation, with selectivity demonstrated against other CDK family members. Linker analysis further showed that the rigid indazolone motif formed via PANAC photoclick chemistry outperformed flexible alkyl linkers.

**Scheme 15:**
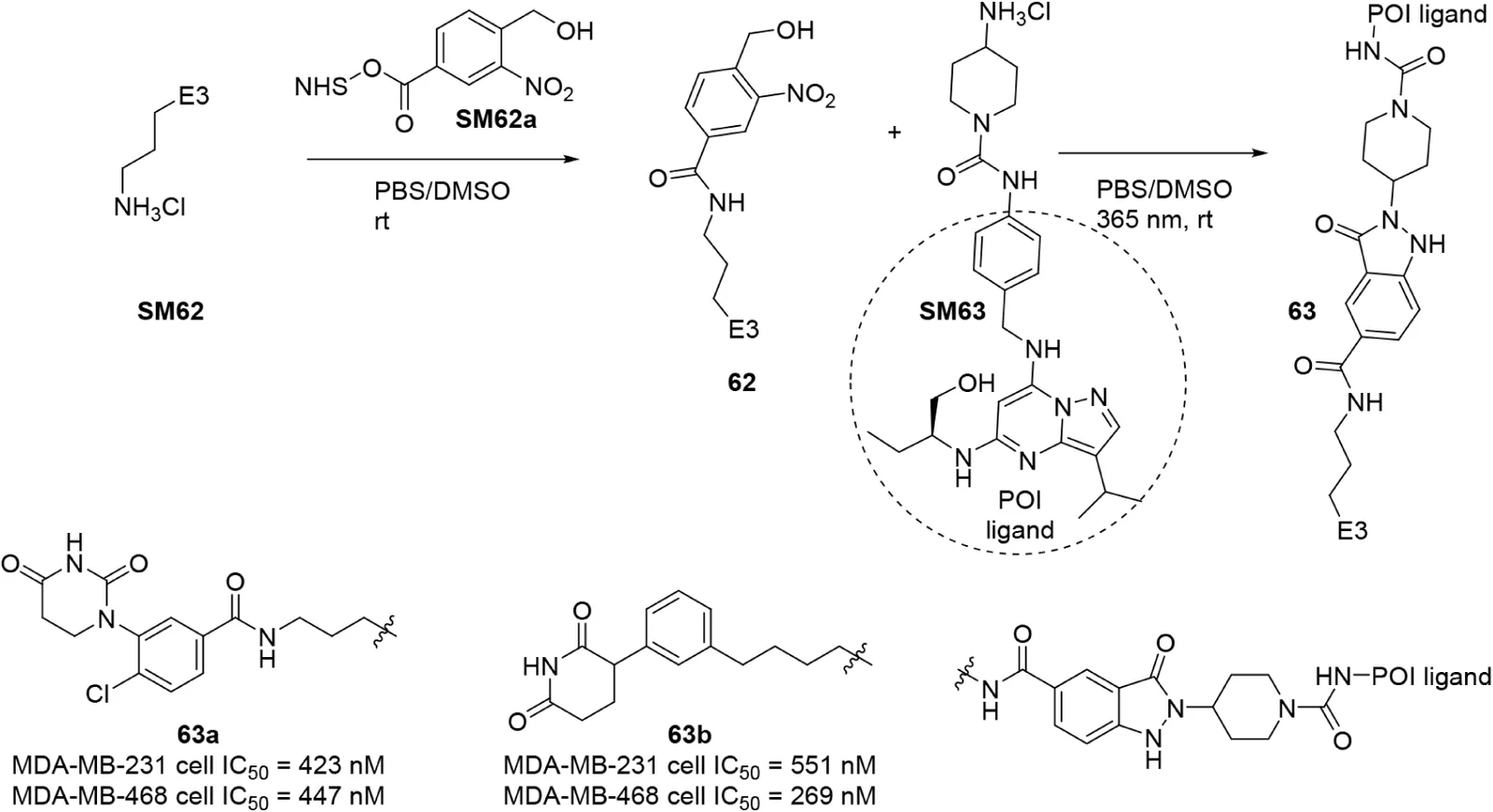
D2B Methodology Implementing a Light-Induced Cyclization Reaction

Holmqvist and colleagues described a plate-based D2B workflow using orthogonally reactive linkers to enable simultaneous variation of POI binders, E3 ligase binders, and linker chemistry toward CNS-active glycogen synthase kinase 3 (GSK3) degraders (Scheme 16).^63^ Libraries were assembled in 96-well plates from benzyl bromide–functionalized E3 ligase binders **SM64** (primarily CRBN, plus one VHL), azide-tagged GSK3 inhibitors **64**, and 12 secondary-amine linkers (**SM64a**) using a sequential S_N_2 alkylation (DMF/triethylamine, 24 h) followed by CuAAC (CuSO_4_/sodium ascorbate/THPTA, 24 h), with all liquid handling automated on an Opentrons OT-2. Incorporation of a basic linker motif enabled plate-based SCX cleanup prior to biology. Eluted products were dried, reconstituted in DMSO, and analyzed by LC–MS. Across two library plates, 147 PROTACs (**65**) were detected, with 103 showing >50% UV purity; failures were mainly associated with α-bromoacetamides or spirocyclic linkers. Crude products were screened in GSK3β-HiBiT knock-in HEK293 cells, enabling full dose–response degradation curves after 24 h. Dozens of active degraders were identified, and six hits were resynthesized and purified, all retaining activity with DC_50_ ≤ 20 nM, including one subnanomolar degrader. Crude-versus-pure rank order was largely preserved, and live-cell kinetics showed near-complete GSK3β loss within ∼2 h at low nanomolar doses. Mechanistic studies confirmed CRBN-dependent degradation, rescue by proteasome and neddylation inhibitors, and selective degradation of GSK3α/β with excellent proteome-wide selectivity. Two optimized leads (**65a** and **65b**) emerged, with **65a** with its better ADME/PK profile advancing to in vivo profiling. Overall, this study demonstrated that orthogonal linker chemistry combined with plate-based SCX cleanup enables rapid D2B assembly, screening, and optimization, delivering probe-quality CNS-active degraders directly from the initial library.

**Scheme 16:**
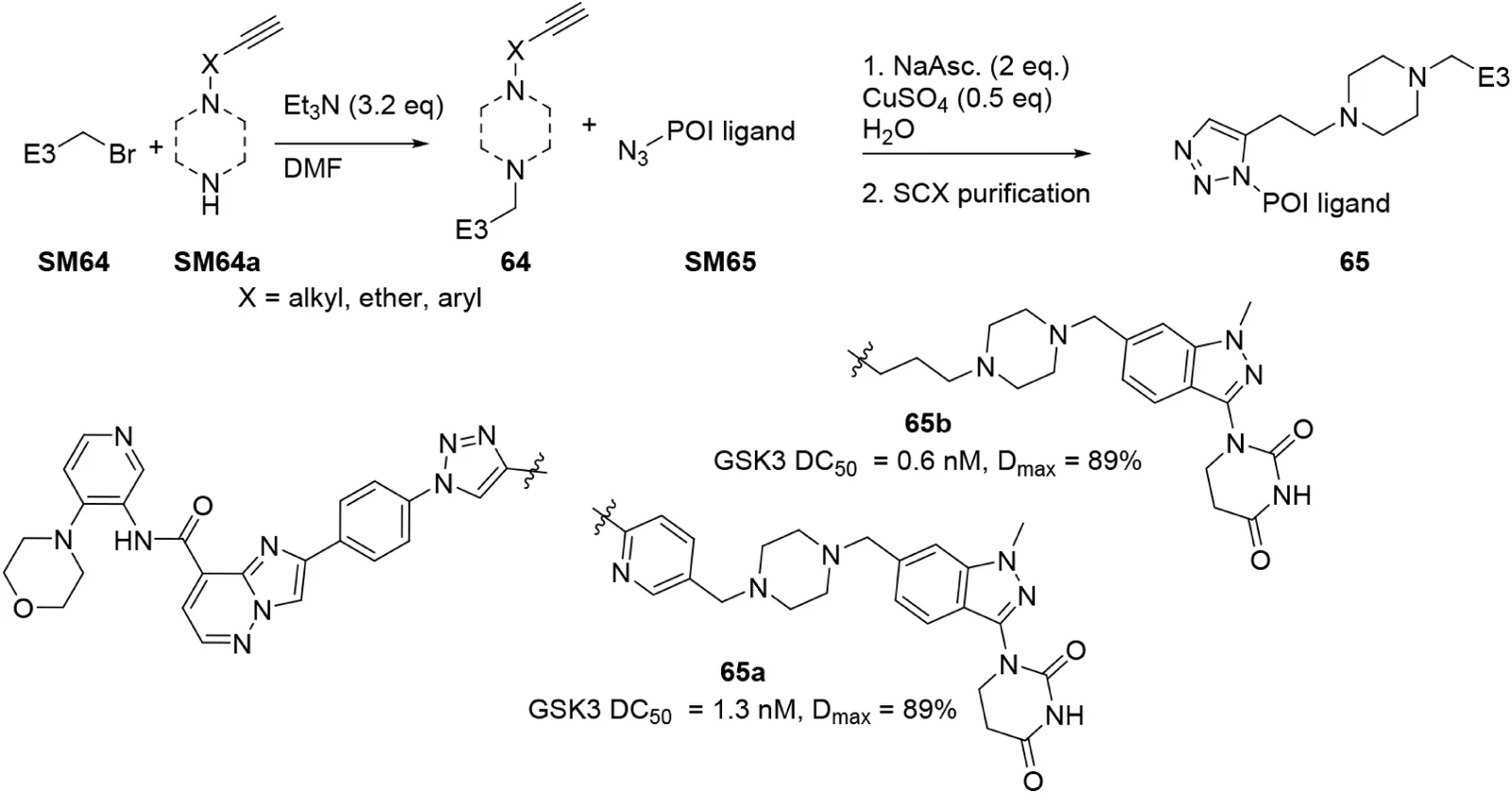
Sequential Alkylation and CuAAC Reactions in a D2B Workflow for the Discovery of Triazole-Based GSK3 PROTACs

Shaum and coworkers extended D2B to the discovery of chemical inducers of proximity (CIPs) and molecular glues by combining high-throughput chemistry (HTC) with direct cellular screening.^64^ Starting from the ENL YEATS-domain ligand **28a**, an iminosulfur oxydifluoride-functionalized analogue **SM31** was diversified by SuFEx click chemistry with 3,163 commercially available primary and secondary amines in 384-well plates (10 μL, 2 mM, 1:1 DMSO/PBS, 37 °C, 24 h), enabling direct testing of crude reaction mixtures in HiBiT-based cellular degradation assays without purification (Scheme 7F). Primary screening identified 93 hits, which were resynthesized and counter-screened against BRD4–HiBiT to eliminate false positives, ultimately yielding the selective ENL degrader **31**. The platform was subsequently applied to the BET inhibitor JQ1, leading to the discovery of potent CIPs, with **32** recruiting the previously unexplored Skp1–Cullin–F-box (SCF) E3 ligase FBXO3 (SCF^FBXO3^) to selectively degrade BRD4. Extensive biochemical, genetic, SPR, crystallographic, and cryo-EM studies demonstrated that both compounds function as molecular glues by stabilizing composite protein–ligand interfaces rather than through classical bifunctional PROTAC design. This work highlights the unique suitability of D2B for the discovery of chemical inducers of proximity, where the ability to synthesize and directly screen thousands of crude reaction products greatly increases the likelihood of identifying the rare active compounds that drive novel proximity-based pharmacology.

Targeted protein degradation arguably represents one of the most compelling applications of D2B. Unlike conventional small-molecule optimization, degrader discovery remains only partially amenable to rational design, as productive degradation depends on a complex interplay between linker composition, exit-vector orientation, ternary complex formation, and E3 ligase selection. Consequently, the ability of D2B platforms to rapidly synthesize and evaluate hundreds of analogues provides a substantial advantage over traditional design–make–purify–test workflows. At present, however, the used chemical reactions remain dominated by a limited number of highly robust ligation reactions, particularly amide coupling and CuAAC, while only a few studies have explored alternative transformations such as reductive amination, S_N_Ar, multicomponent reactions, or photoclick chemistry. Likewise, despite the large number of human E3 ligases, most reported D2B campaigns continue to rely on the well-established cereblon and von Hippel–Lindau ligases, leaving much of the degradable proteome unexplored. In this context, D2B-guided molecular glue discovery may represent an even greater opportunity than conventional PROTAC optimization, as productive glue interactions remain exceptionally difficult to predict by structure-based or computational approaches. The combination of broad chemical diversification with direct biological evaluation therefore provides an efficient strategy for uncovering chemical inducers of proximity and expanding the accessible space of proximity-based pharmacology. As reaction diversity and the repertoire of recruitable E3 ligases continue to expand, D2B is well positioned to play a central role in the discovery of next-generation proximity-inducing therapeutics.

### D2B Approaches Developing Macrocycles

Macrocycles occupy a unique region of chemical space between small molecules and biologics, defined by rings of at least 12 heavy atoms.^65^ Their conformationally constrained yet adaptable structures often enable high binding affinity and selectivity, particularly for challenging targets lacking well-defined binding pockets.^66^ Cyclic peptides, formed by head-to-tail or side-chain-driven cyclization of linear sequences, represent a prominent subclass in which amino acids encode both structural and functional complexity through backbone amides and diverse side chains.^67^ To date, more than 60 macrocyclic drugs have been approved, and despite frequently exceeding Lipinski’s rule of five, a significant fraction remains orally bioavailable.^65^ At the same time, macrocycle discovery is synthetically demanding, as ring-closing efficiency and linker design are critical challenges.^65^ Direct-to-biology strategies offer an attractive solution by enabling rapid generation and screening of large macrocyclic libraries, where crude products are tested directly and only validated hits are resynthesized, making exploration of this complex chemical space significantly more efficient.

Kale and coworkers have developed a series of plate-based macrocycle discovery platforms enabling rapid identification and optimization of bioactive cyclic peptides through high-yield thiol-based cyclization and combinatorial diversification (Table 1).^68^ Their initial strategy employed cysteine addition to bis-electrophiles followed by intramolecular N-terminal amine closure, achieving up to ∼96% cyclization efficiency and enabling direct crude screening. Seven optimized bis-electrophiles were used to generate libraries up to 8,988 members in a 384-well format. Crude macrocycles were screened against trypsin-like serine proteases (trypsin, thrombin, human kallikrein 5 (KLK5)) using fluorogenic AMC substrates, identifying inhibitors (**66**) with *K*_i_ values down to 42 nM, while X-ray structures revealed unexpected linker-derived binding motifs. Chemical diversity was expanded via sequential *N*-alkylation of bromoacetamide peptides with external amines, TCEP-mediated cysteine deprotection, and final bis-electrophile cyclization.^69^ This three-step sequence (alkylation → deprotection → cyclization; ∼86% overall yield) yielded 3,780 macrocycles, which were screened directly in a fluorescence-based thrombin activity assay (AMC substrate, assumed full conversion). Several potent inhibitors were identified, including a lead (**67**) with a *K*_i_ of 4.2 nM. This example highlights the power of combinatorial amine and linker SAR. The workflow was subsequently miniaturized using acoustic droplet ejection, enabling picomole-scale alkylation and cyclization to generate 2,700-member libraries targeting the MDM2–p53 protein–protein interaction.^70^ Crude macrocycles were screened using a fluorescence polarization displacement assay, in which inhibition of MDM2 binding to a fluorescent p53-derived reporter peptide produced a signal change. Fourteen hits were identified, including submicromolar binders, and these compounds (**68a**, **68b**) were resynthesized and validated by surface plasmon resonance (SPR), showing strong agreement between crude and purified activities.

**Table 1:**
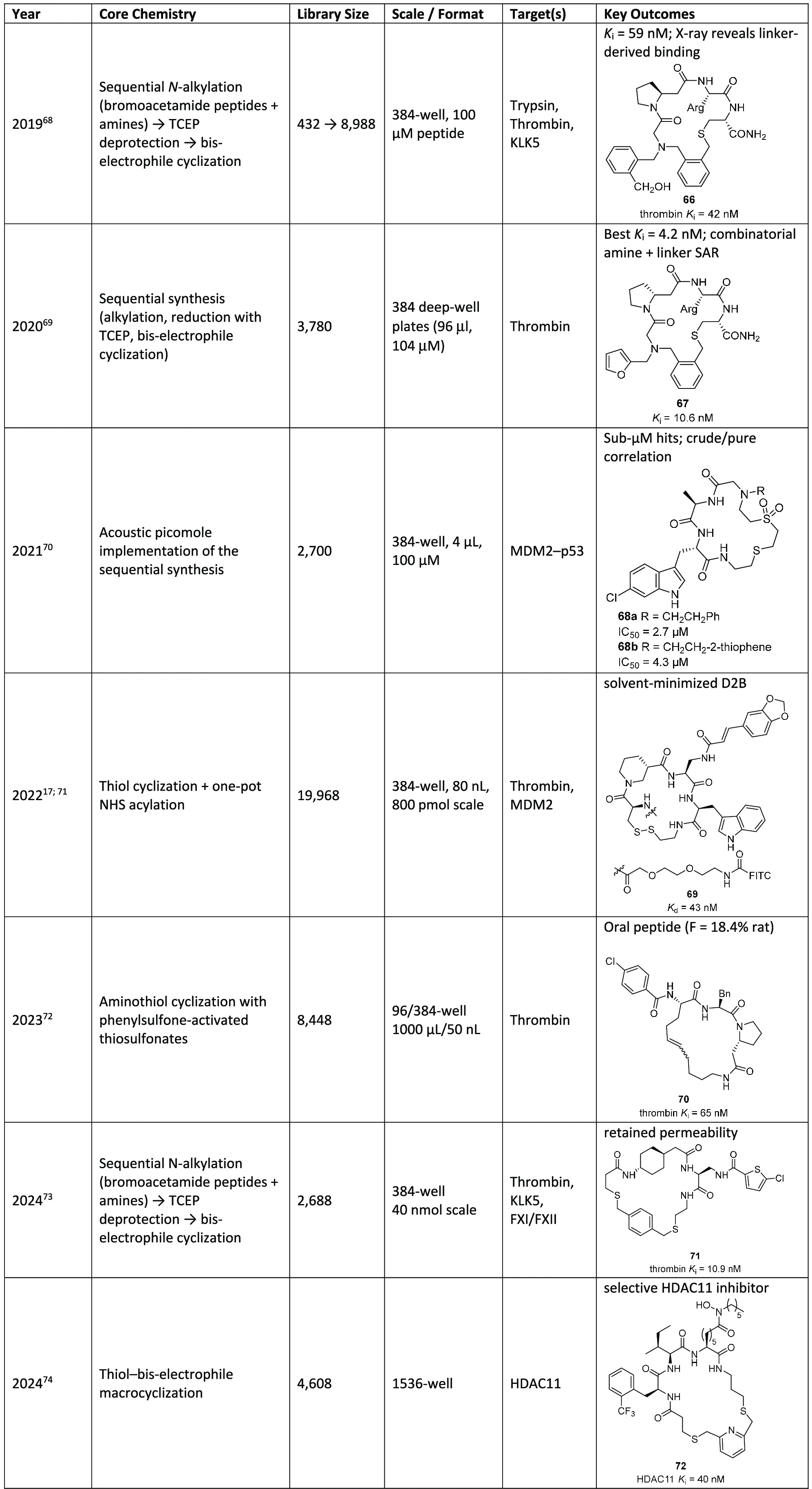
Overview of D2B Macrocycle Discovery Platforms and Macrocyclization Chemistries

In 2022, peripheral diversification was introduced by performing ultralow-volume amide coupling directly on preformed macrocycles (800 pmol in 80 nL DMSO) using carboxylic acids (4 equiv) and HBTU, where replacing DIPEA with DABCO restored high coupling yields, and excess activated acid was quenched with Tris–Cl.^71^ Nearly 20,000 macrocyclic amides were generated and screened directly for thrombin inhibition (fluorogenic AMC assay) and MDM2–p53 disruption (FP), yielding hits (e.g., **69**) in both campaigns with *K*_i_ ≈ 40 nM.

More recent iterations addressed developability. Combining thiol cyclization with one-pot acylation and replacing oxidation-prone thioethers by ring-closing metathesis–derived hydrocarbon linkers enabled construction of an 8,448-member library screened directly against thrombin using a fluorogenic assay, followed by parallel profiling for proteolytic stability, PAMPA permeability, and microsomal clearance, ultimately delivering orally bioavailable cyclic peptides (**70**) (*K*_i_ = 65 nM; *F* = 18.4% in rats).^72^ Finally, diversification of the cyclization chemistry itself using aminothiol building blocks activated via phenylsulfone thiosulfonates produced 2,688 membrane-permeable macrocycles, which were screened directly after synthesis against five serine proteases (thrombin, plasma kallikrein, KLK5, Factor XI and XII (FXI, FXII)) using AMC substrates in a fully automated campaign. This large study yielded nanomolar inhibitors (**71**) (best *K*_i_ = 10.9 nM) while retaining cellular permeability.^73^

Most recently, the platform was extended to zinc-dependent targets through the de novo discovery of selective macrocyclic HDAC11 inhibitors.^74^ A protected hydroxamic acid–containing amino acid building block enabled incorporation of zinc-chelating motifs into the macrocycles. In a 1536-well format, 768 thiol-containing peptides were cyclized with bis-electrophiles using automated liquid handling and acoustic dispensing, generating 4,608 macrocycles. Crude products were screened directly in a fluorogenic HDAC11 assay, identifying 12 hits. Resynthesized compounds revealed a selective inhibitor **72** (*K*_i_ = 40 nM), highlighting the adaptability of macrocycle D2B platforms to metal-dependent enzymes.

The current body of work demonstrates that D2B is particularly well suited to macrocycle discovery, where the high synthetic complexity of macrocyclic compounds makes rapid screening of crude libraries especially valuable. At the same time, the field remains comparatively narrow, with all reported examples originating from a single research group and relying on peptide-derived scaffolds assembled through highly efficient thiol-based cyclization chemistries. These modular platforms provide exceptional robustness and high reaction conversion, making them ideally suited for D2B workflows, but they also benefit from the inherent accessibility of peptide synthesis. Whether similar success can be achieved with structurally more diverse, nonpeptidic macrocycles requiring more demanding ring-forming reactions remains to be established. Future advances should therefore focus on expanding the repertoire of D2B-compatible macrocyclization chemistries and scaffold classes while maintaining the robustness and high conversion necessary for direct biological screening.

### D2B in Covalent Fragment Expansion

Fragment-based drug discovery enables efficient exploration of chemical space using low-molecular-weight compounds that bind with high ligand efficiency, including to shallow or cryptic binding sites.^75^ However, their weak affinities require rapid iterative optimization. D2B strategies are well suited to this process, as they enable parallel fragment expansion and direct screening of crude products using biophysical methods such as SPR, NMR, X-ray crystallography, or mass spectrometry. This integration accelerates SAR development and the transition from weak fragments to higher-affinity leads while maintaining throughput and reducing resource demand.

Kooij and coworkers reported an acoustically dispensed, plate-based D2B amide-coupling platform.^76^ A cyanimide-focused library was assembled by combining 16 cyanimide amines **SM73a** with 471 carboxylic acids **SM73** yielding 7,536 compounds **73** (Scheme 17A). Coupling reagents were first screened in vials (HBTU, HCTU, HATU, PyBOP/NMM; EDC/HOBt, DIC/HOBt, EDC/DMAP, DIC/DMAP), identifying DIC/HOBt (5 equiv each) in DMSO as optimal for high conversion and low byproduct formation. Reactions were run at 10 μL in 384-well plates and miniaturized to 2.5 μL in 1536-well plates using microplate dispensing for DIC/HOBt and acoustic transfer for remaining components, achieving ∼85% success by LC–MS sampling. Residual reagents did not interfere with assays. 10-fold dilution daughter plates were prepared, and crudes were screened directly at 1.25 μM against 12 ubiquitin and ubiquitin-like proteases using a fluorescence assay, enabling rapid selectivity profiling. Selective ubiquitin-specific peptidase 18 (USP18) inhibitors were identified. The purified hits correlated well with data measured on crude reaction mixtures. Focused optimization delivered **73a**, a covalent USP18 inhibitor (35 nM) with high selectivity across 41 DUBs and confirmed cellular engagement.

**Scheme 17:**
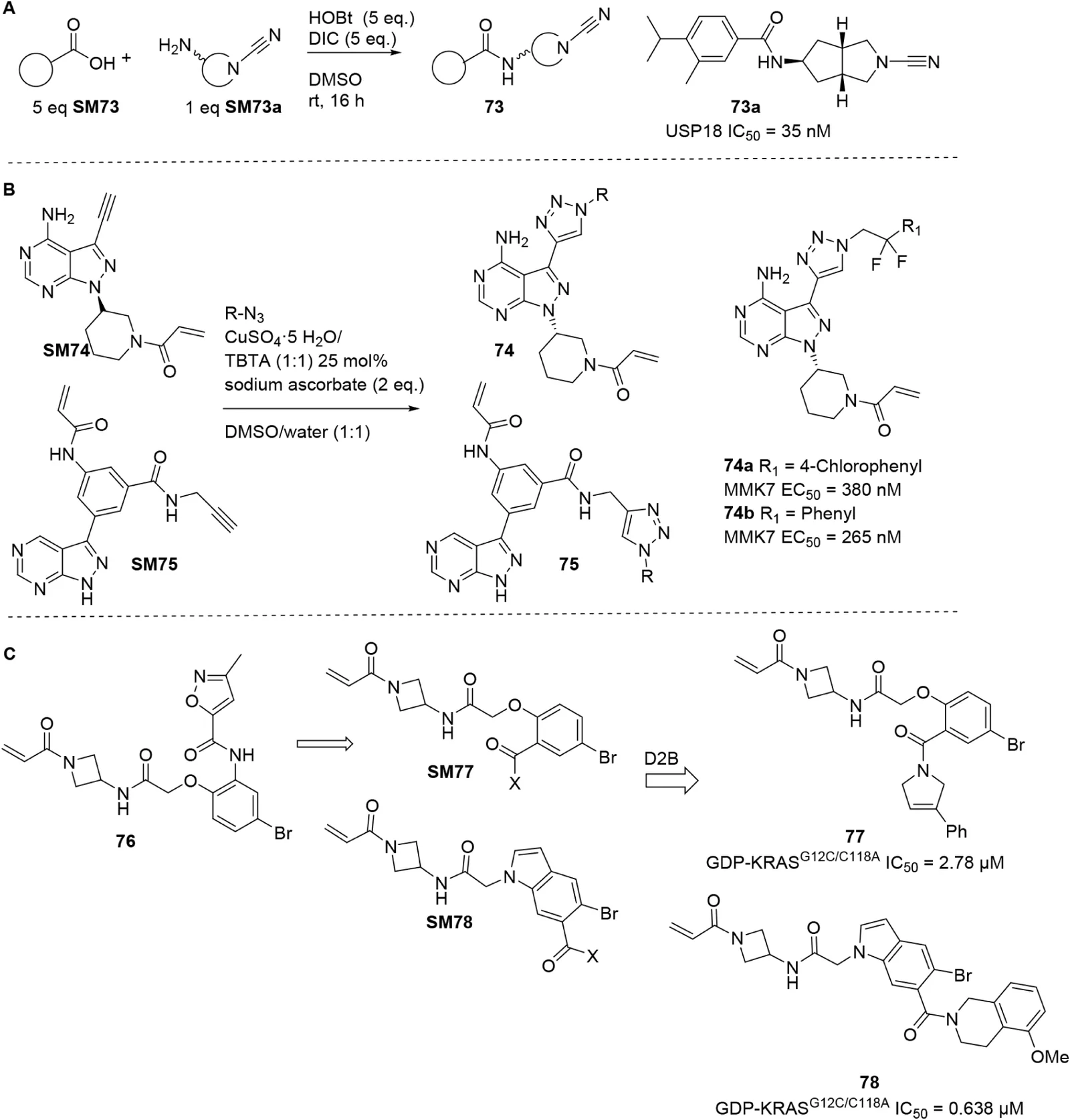
D2B Workflows for Covalent Fragment Drug Discovery[Fn sch17-fn12]

a (A) Amide coupling-based library targeting USP18. (B) CuAAC-enabled D2B workflow for the discovery of MKK7 covalent ligands. (C) Chemotype evolution platform for the discovery of KRAS ligands.

Focusing on covalent inhibitor screening against mitogen-activated protein kinase kinase (MKK7), Gehrtz and coworkers developed a D2B approach based on CuAAC, a mild and electrophile-tolerant reaction.^77^ Two inhibitor scaffolds bearing an alkyne functionality (**SM74**, **SM75**) and an acrylamide warhead were combined with a commercially available azide library comprising 448 members (Scheme 17B). Reactions were carried out in DMSO/water (1:1) using CuSO_4_, TBTA, and sodium ascorbate at a 40 nmol scale in a total volume of 2.5 μL. Using an acoustic droplet ejection robotic system, the reactions were performed in 384-well plates, and the crude reaction mixtures (**74**, **75**) were directly evaluated using an in-cell Western assay in U2OS cells. Target engagement was assessed by immunostaining of phosphorylated JNK induced by osmotic stress following sorbitol treatment. Hits were identified for both inhibitor scaffolds, subsequently resynthesized, purified, and characterized in greater detail. Crystallographic studies of the most potent compounds revealed their binding modes, showing that the first scaffold class acted as a type-I kinase inhibitor and that the triazole moiety did not participate in binding, regardless of the binding orientation. Cellular potency studies demonstrated that the D2B campaign yielded compounds more potent (**74a** and **74b**) than the parent scaffold **SM74**, whereas reduced potency was observed for scaffold 2 (**SM75** vs **75**).

To identify inhibitors of Kirsten Rat Sarcoma KRAS^G12C^, Shin and coworkers developed a D2B strategy termed Chemotype Evolution,^78^ in which electrophilic fragments are rapidly diversified and screened.^79^ Five anchor molecules bearing both a reactive handle and a covalent electrophilic warhead were prepared (Scheme 17C). Warhead reactivity was evaluated in a glutathione assay and found to be comparable to that of clinically used covalent kinase inhibitors. Using nanogram-scale coupling of fragments (experimental details are not included) to the reactive handle, 3,300 crude compounds were generated and screened with a concentration of 2 μM of protein across multiple assays. These assays included a thiol-reactive probe assay, a RAF-coupled nucleotide exchange assay, and intact-protein mass spectrometry. Eight hits were identified, and resynthesis and purification of the leading compound **76** enabled cocrystallization with KRAS^G12C^, confirming its covalent binding mode. In a second D2B cycle, the initial hit was elaborated by fragment extension via amide coupling along two exit vectors (**SM77**, **SM78**), generating a library of ∼5,500 amide-containing analogues. Screening identified 71 hits. From the phenol-based anchor series, **77** emerged as the most potent, retaining the binding mode of **76** while forming additional favorable interactions, as shown by X-ray crystallography. From the indole-based series, the top hit displayed two different conformations in cocrystal structures. Both hits were further characterized in functional assays, and **78** was selected for continued optimization. Although the final compound was not advanced as a drug candidate, it proved valuable as a tool compound for revealing a previously unrecognized subsite on KRAS^G12C^.

To address rising bacterial antibiotic resistance, Danti et al. explored peptide nucleic acid (PNA)–based antisense antibiotics, which inhibit bacterial growth by blocking mRNA translation and essential protein synthesis.^80^ PNAs feature a neutral *N*-(2-aminoethyl)­glycine backbone in place of the phosphate–ribose scaffold, with nucleobases attached directly and lacking a sugar moiety. Because PNA efficacy is difficult to predict, the authors implemented a high-throughput D2B strategy to enable systematic screening and optimization. Targeting focused on the translation initiation region (TIR) of essential bacterial mRNAs, using a tiling approach in which partially overlapping antisense sequences are shifted by a few nucleotides to achieve high-density coverage of potential binding sites. PNA synthesis was performed using Fmoc-based solid-phase peptide synthesis on highly derivatized cellulose discs, employing commercially available protected monomers at low concentration (100 mM). Coupling was achieved using PyOxim/DIPEA or OxymaPure/DIC, with the latter providing coupling efficiencies above 97%. Automated synthesis was carried out on a MultiPep RSi robot. Using this platform, 1,536 PNA–peptide conjugates (dPNAs) were synthesized at a 100 nmol scale, with commercially available PNAs (cPNAs) serving as references. The crude dPNAs generally exhibited higher minimal inhibitory concentrations (MICs) than purified cPNAs and showed 4- to 8-fold reduced potency. Antibacterial screening was conducted in 384-well plates against uropathogenic *E. coli* strain 536 (UPEC 536). The initial target was the TIR of *acpP* mRNA, and PNAs were fused directly to the (KFF)_3_K delivery sequence to ensure cellular uptake. Screening of 6–11-mer *acpP*-targeting PNAs identified 8–10-mers as optimal for growth inhibition. The disc-based synthesis and tiling platform was subsequently applied to additional essential genes involved in cell division, DNA replication, and repair. Potent growth inhibition was observed for three targets, intermediate activity for three others, and no detectable activity for two previously untested mRNAs. Overall, the established tiling and D2B synthesis platform markedly accelerated PNA hit identification and optimization.

Wilders and coworkers established a covalent fragment–based D2B platform that combines intact-protein mass spectrometry with plate-based amide coupling to rapidly optimize electrophilic fragments across multiple cysteine-containing targets. The approach was first demonstrated against the SARS-CoV-2 main protease (M^pro^).^81^ An intact-protein LC–MS screen of 219 chloroacetamide fragments (5 μM fragment, 0.5 μM M^pro^, 16 h, 4 °C) identified six covalent hits (e.g., **80**) (>75% labeling). To enable rapid SAR, an NHS-activated 2-chloroacetyl handle **79** was used for plate-based amide coupling (DMSO, RT, 1 h, 1.2 equiv ester, 3 equiv DIPEA) to generate analogues as compound **81** (Scheme 18A). A second D2B campaign explored SAR using a focused 193-member morpholine-derived amine library selected via small-world analysis, yielding 14 compounds with >75% labeling and up to 10-fold improved inhibition while retaining activity against a clinically relevant M^pro^ mutant. The workflow was subsequently adapted to a separate noncovalent optimization campaign (**82**) performed in two rounds: 146 carboxylic acids, selected by small-world analysis from the GSK compound collection, were coupled via HATU-mediated amide coupling (1.5 equiv HATU, 3 equiv NMM, DMSO, 60 °C, 1 h, 384-well plates, 80 μL). Crude products were screened in a FRET assay, yielding seven hits (**83**, best pIC_50_ = 5.6). A second focused set of 72 acids further refined SAR and produced an improved inhibitor **84** with pIC_50_ = 6.7.

**Scheme 18:**
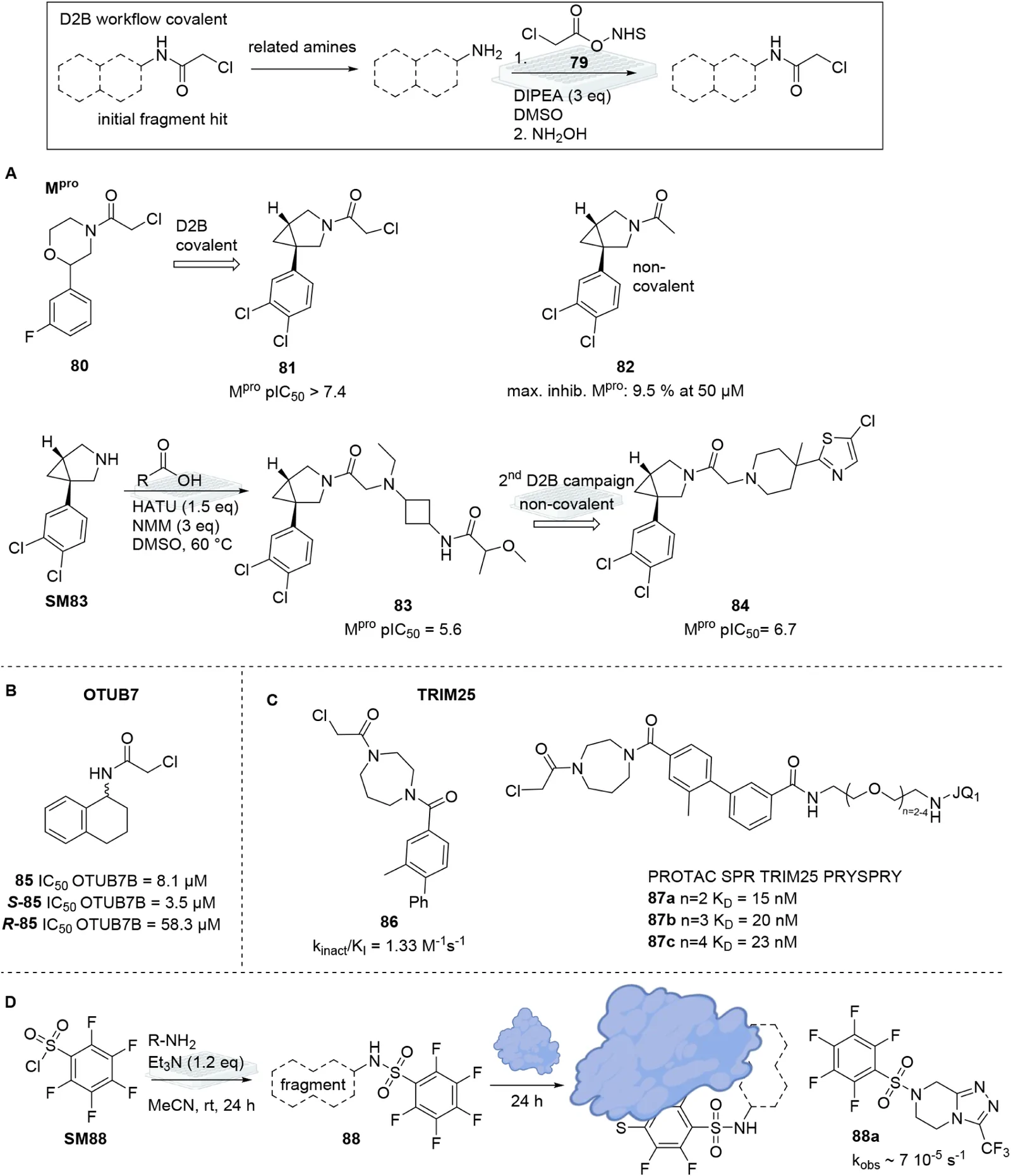
D2B Workflows Employing Reactive Moieties for Covalent Ligand Discovery[Fn sch18-fn13]

a (A) Discovery of covalent and noncovalent ligands targeting the SARS-CoV-2 main protease. (B) Discovery of OTUB7 ligands. (C) Identification of a covalent TRIM25 ligand and its subsequent conversion into PROTACs. (D) Covalent ligand discovery targeting AurkA.

This platform was then extended to chemoproteomics-guided discovery of ovarian tumor (OTU) deubiquitinases (DUBs).^82^ Using a biotinylated ubiquitin vinyl sulfone activity-based probe, 227 chloroacetamide fragments were screened in HEK293T lysates, identifying interactions with multiple DUBs. Seven fragments targeting five OTUs (OTUD4, OTUD5, OTUD7A/B, and ZRANB1) met selectivity criteria. Fragment expansion employed the same NHS-chloroacetyl amide coupling in 384-well plates (40 μL, 1 h) with hydroxylamine quenching. From 351 amines, LC–MS analysis showed >50% conversion in 136 wells. Crude products were screened by intact-protein MS against recombinant OTU enzymes, yielding 21 validated ligands; enantiomer resolution revealed a 13-fold activity increase for the *S*-enantiomer of compound **85** with limited off-target cysteine engagement (Scheme 18B).

A related workflow was subsequently applied to the PRYSPRY domain of tripartite motif-containing protein 25 (TRIM25).^83^ An intact-protein LC–MS fragment screen of 221 chloroacetamide fragments (50 μM) against recombinant TRIM25 PRYSPRY (0.25 μM, 4 °C, 24 h) identified eight covalent hits. Three prioritized fragments were expanded via NHS-ester amide coupling in 384-well plates, generating three focused libraries (83, 212, and 186 members; 481 total). Productive coupling (>50% conversion) was observed for 69/83, 102/212, and 132/186 reactions. Screening by intact-protein LC–MS identified ligands with 6- to 18-fold improved *k*_inact_/*K*_i_ while maintaining comparable glutathione reactivity (∼1.9 h). Purified compound **86** selectively modified Cys498, engaged TRIM25 in THP-1 cells, and its binding mode was confirmed by a 1.8 Å crystal structure (Scheme 18C). Incorporation into JQ1-based heterobifunctional molecules (**87a**-**87c**) enabled ternary complex formation with BRD4 and TRIM25-dependent BRD4 ubiquitination, demonstrating direct repurposing of a newly liganded E3 ligase.

Chesti and coworkers developed a D2B platform using sulfonylation as a connective reaction to generate reactive fragment libraries (Scheme 18D).^84^ A set of 96 amines was reacted with pentafluorobenzenesulfonyl chloride (SM88a) in a custom PTFE 96-well plate equipped with 750 μL borosilicate glass vials (20 mM, MeCN, 90 μL, rt, 24 h) using triethylamine (1.2 equiv); anilines and heteroaryl amines were excluded due to poor reaction cleanliness, and sulfonylation reactions were found to be incompatible with DMSO as solvent. After solvent removal, crude products (88) were dissolved in DMSO to give 20 mM stock solutions. Crude mixtures were diluted (200 μM) and screened directly against AurkA (2 μM) using intact-protein LC–MS after 24 h. To assess selectivity, fragments were also screened against NIMA-related kinase 7 and ubiquitin-conjugating enzyme E2 D2. Hits (Z score> 2.5) were identified and validated, with kinetic analysis showing measurable labeling rates (best hit 88a). LC–MS/MS mapping revealed modification of Cys247 and Cys290, including the typically buried Cys247, highlighting access to cryptic binding sites.

The examples presented in this section illustrate that D2B is particularly powerful whenever rapid iterative analogue synthesis is required to refine an initial chemical hypothesis. Whether applied to fragment elaboration, covalent ligand optimization, or focused analogue generation, the combination of modular, high-yielding transformations with direct biological screening enables rapid exploration of local SAR while minimizing synthetic effort. In addition, orthogonal techniques such as intact-protein mass spectrometry, X-ray crystallography and SPR provide efficient validation of crude-screening results and mechanistic insight into target engagement. At present, however, many reported workflows remain rely on a relatively limited set of robust ligation reactions, such as amide coupling and CuAAC. Future developments should therefore focus on expanding the synthetic toolbox and enabling more general diversification across a broader range of target classes, scaffold types, and electrophilic modalities while maintaining the robustness required for direct biological screening.

## Conclusions

The studies highlighted in our perspective review demonstrate that D2B has evolved into a rapid medicinal chemistry ligand optimization strategy. Thus, D2B has shifted early hit discovery from a sequential design–make–test paradigm toward a highly efficient parallel synthesis workflow with direct (biological) evaluation. By combining robust chemistry, miniaturized synthesis, automation, and increasingly diverse biological readouts, D2B substantially shortens the design–make–test cycle while reducing synthetic effort. Nevertheless, current D2B workflows remain primarily focused on early discovery, and promising hits generally require conventional medicinal chemistry optimization to improve potency, selectivity, and in some cases, pharmacokinetic properties.

Our analysis of reported D2B campaigns revealed that the field is currently defined more by reaction robustness than synthetic diversity (Figure 3). Nearly half of all reported workflows rely on amide bond formation (42%), followed by click chemistry (25%) and nucleophilic substitution (16%), whereas transition-metal-catalyzed cross-couplings, condensations, and other modern transformations remain rare (Figure 3A). This distribution reflects the central requirement that D2B reactions must proceed with consistently high conversion while remaining compatible with direct biological evaluation of crude reaction mixtures. Although several recent studies have demonstrated Suzuki couplings, Buchwald–Hartwig aminations, Rh-catalyzed late-stage hydrogenation, and emerging C–H functionalization reactions, the wider medicinal chemistry toolbox remains largely unexplored in D2B. Future efforts should therefore focus on expanding robust cross-coupling methodologies, visible-light photoredox and metallaphotoredox catalysis, electrochemical transformations, and multicomponent reactions, enabling increasingly complex late-stage diversification while maintaining assay compatibility.

**Figure 3:**
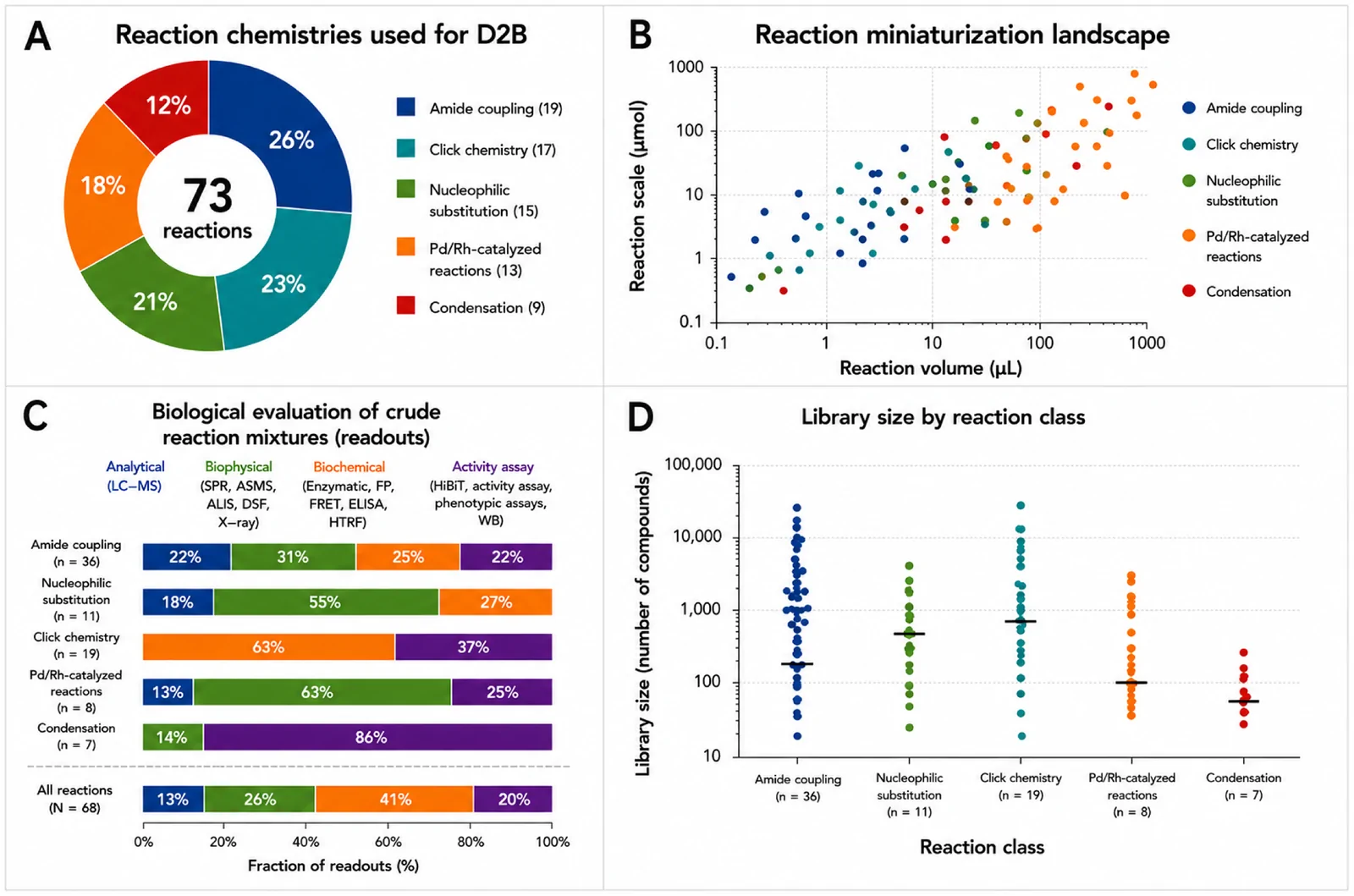
Graphical summary of the D2B workflows discussed in this perspective. (A) Distribution of reaction classes. (B) Reaction scale and volume. (C) Biological assay readouts. (D) Library sizes.

A similar balance between robustness and information content is evident from the distribution of biological readouts (Figure 3C). Most reported D2B campaigns continue to rely on biochemical and biophysical assays, reflecting their quantitative nature, tolerance toward residual reaction components, and straightforward implementation in high-throughput formats. In contrast, cellular assays remain less common because they impose substantially higher demands on reaction cleanliness and assay compatibility. Nevertheless, their ability to simultaneously capture permeability, intracellular target engagement, toxicity, and other physiologically relevant properties suggests that they will play an increasingly important role as D2B-compatible chemistries continue to improve.

Reaction miniaturization has similarly expanded over several orders of magnitude (Figure 3B), from conventional microliter-scale plate chemistry to nanoliter acoustic dispensing and subnanomole synthesis. While most D2B campaigns are currently performed in 1 to 100 μL volumes, recent examples demonstrate reliable chemistry at picomolar to nanomolar scale, dramatically reducing material consumption and facilitating access to increasingly large chemical libraries. Interestingly, library size does not correlate directly with reaction scale but instead reflects the maturity and robustness of the underlying chemistry (Figure 3D). Click chemistry and amide coupling routinely support libraries comprising several thousand compounds, whereas more complex transformations, including Pd-catalyzed reactions and condensations, are typically implemented on smaller library scales. This disparity highlights that reaction reliability, rather than miniaturization alone, remains the principal determinant of successful large-scale D2B campaigns. As liquid-handling technologies, acoustic dispensing, and automated reaction platforms continue to evolve, further expansion of both reaction diversity and accessible library size can be anticipated.

An important observation that emerged from this survey was that successful D2B does not necessarily mean the complete absence of workup. Instead, most mature platforms employed only the minimum sample preparation required to ensure assay compatibility, such as solvent exchange, deprotection, reagent quenching, or filtration, while deliberately avoiding chromatographic purification and compound isolation. This pragmatic balance between synthetic simplicity and biological robustness is a recurring feature of successful D2B workflows.

A emerging challenge is data management. As D2B campaigns increasingly generate thousands to tens of thousands of reactions per experiment, standardized recording, storage, and analysis of reaction metadata become essential. Recent developments such as phactor,^85^ which provides machine-readable workflows linking reaction design, automation, analytical data, and biological results together with AI-assisted reaction array design^86^ and machine-learning-guided reaction optimization for highly parallel experimentation,^87^ illustrate how digital infrastructure can transform D2B into a closed-loop discovery workflow. Integration of standardized reaction databases, automated liquid handling, AI-assisted experimental design, and Bayesian optimization should increasingly enable self-improving D2B platforms that iteratively refine both reaction conditions and library design

Despite these challenges, the strong concordance observed between crude and purified compounds across numerous studies demonstrates that D2B has matured into a reliable medicinal chemistry strategy rather than merely a high-throughput screening approach. The continued expansion of the reaction toolbox, improved digital data infrastructure, and tighter integration with machine learning, chemoproteomics, and advanced cellular readouts are expected to further increase its predictive power. Rather than replacing conventional medicinal chemistry, D2B fundamentally shifts where experimental effort is invested, from purification before biological validation to biological validation before extensive synthesis. As chemistry, automation, and computational design continue to converge, D2B is poised to become an integral component of future medicinal chemistry workflows, enabling faster and more resource-efficient exploration of chemical space across both established and emerging therapeutic modalities.

## Abbreviations

ACTs: 2-aryl-5-carboxytetrazoles
ALIS: affinity-selection mass spectrometry
AR: androgen receptor
ASMS: affinity-selection mass spectrometry
BCL6: B-cell lymphoma 6
BRD3: bromodomain-3
BRD4: bromodomain-containing protein 4
B-SPA: Binding-Site Purification of Actives
CAI: human carbonic anhydrase I
CDK2: cyclin-dependent kinase 2
CDK9: cyclin-dependent kinase 9
CDK12: cyclin-dependent kinase 12
CHK1: checkpoint kinase 1
Clint: microsomal intrinsic clearance
CRBN: cereblon CuAAC copper-catalyzed azide–alkyne cycloaddition
CuAAC: copper-catalyzed azide–alkyne cycloaddition
D2B: direct-to-biology
DCAF16: DDB1- and CUL4-associated factor 16
DNPH1: 2′-deoxynucleoside 5′-monophosphate *N*-glycosidase
DsbA: disulfide bond-formation protein A
DUBs: deubiquitinases
ENL: 11–19 leukemia
ER: estrogen receptor
ERK2: extracellular-signal regulated kinase 2
FP: fluorescence polarization
FXI: factor XI
GSK3: glycogen synthase kinase 3
GSPT1: G1 to S phase transition protein 1
HA: influenza hemagglutinin
HDAC: histone deacetylase
HER2: human epidermal growth factor receptor 2
HSP90: heat shock protein 90
HTC: high-throughput chemistry
HTRF: homogeneous time-resolved fluorescence
KLK5: human kallikrein 5
KRASG12C: Kirsten rat sarcoma G12C mutant
LC–CAD–MS: liquid chromatography–charged aerosol detection–mass spectrometry
LLE: lipophilic ligand efficiency
MDM2: mouse double minute 2 homologue
MICs: minimal inhibitory concentrations
MKK7: mitogen-activated protein kinase kinase 7
MK2: MAPKAP kinase 2
Mpro: SARS-CoV-2 main protease
MST: microscale thermophoresis
NHS: *N*-hydroxysuccinimide
NMI: 1-methylimidazole
NMP: *N*-methyl-2-pyrrolidon
NNRTIs: non-nucleoside reverse transcriptase inhibitors
OPA: *ortho*-phthalaldehyde
ORS: off-rate screening
OUT: ovarian tumor
PANAC: *p*-nitrobenzyl alcohol cyclization
PDHK: pyruvate dehydrogenase kinase
PIN1: peptidyl-prolyl *cis*–*trans* isomerase NIMA-interacting 1
PHIP­(2): Pleckstrin Homology Domain-Interacting Protein 2
PNA: peptide nucleic acid
POI: protein of interest
PRDX1: peroxiredoxin-1
REFiL: Rapid Elaboration of Fragments into Leads
RIPK2: receptor-interacting serine/threonine-protein kinase 2
SAM: *S*-adenosylmethionine
SCF: Skp1–Cullin–F-box
she: soluble epoxide hydrolase
SHP2: Src homology region 2 tyrosine phosphatase 2
SuFEx: sulfur–fluoride exchange
TBTA: tris­(benzyltriazolylmethyl)­amine
TCEP: tris­(2-carboxyethyl)­phosphine
tDHU: tolyldihydrouracil
TIR: translation initiation region
TrmD: tRNA m^1^G37 methyltransferase
TRIM25: tripartite motif-containing protein 25
ultraHTE: ultrahigh-throughput experimentation
USP18: ubiquitin-specific peptidase 18
VHL: von Hippel–Lindau protein
